# Endocytosis-like DNA uptake by cell wall-deficient bacteria

**DOI:** 10.1038/s41467-022-33054-w

**Published:** 2022-09-22

**Authors:** Renée Kapteijn, Shraddha Shitut, Dennis Aschmann, Le Zhang, Marit de Beer, Deniz Daviran, Rona Roverts, Anat Akiva, Gilles P. van Wezel, Alexander Kros, Dennis Claessen

**Affiliations:** 1 Institute of Biology, Leiden University, Sylviusweg 72, 2333 Leiden, The Netherlands; 2 Department of Supramolecular and Biomaterials Chemistry, Leiden Institute of Chemistry, Leiden University, Einsteinweg 55, 2333 Leiden, The Netherlands; 3 Electron Microscopy Center, Radboudumc Technology Center Microscopy, Nijmegen, The Netherlands

**Keywords:** Cellular microbiology, Endocytosis, DNA metabolism

## Abstract

Horizontal gene transfer in bacteria is widely believed to occur via conjugation, transduction and transformation. These mechanisms facilitate the passage of DNA across the protective cell wall using sophisticated machinery. Here, we report that cell wall-deficient bacteria can engulf DNA and other extracellular material via an endocytosis-like process. Specifically, we show that L-forms of the filamentous actinomycete *Kitasatospora viridifaciens* can take up plasmid DNA, polysaccharides (dextran) and 150-nm lipid nanoparticles. The process involves invagination of the cytoplasmic membrane, leading to formation of intracellular vesicles that encapsulate extracellular material. DNA uptake is not affected by deletion of genes homologous to *comEC* and *comEA*, which are required for natural transformation in other species. However, uptake is inhibited by sodium azide or incubation at 4 °C, suggesting the process is energy-dependent. The encapsulated materials are released into the cytoplasm upon degradation of the vesicle membrane. Given that cell wall-deficient bacteria are considered a model for early life forms, our work reveals a possible mechanism for primordial cells to acquire food or genetic material before invention of the bacterial cell wall.

## Abstract (web summary)

Horizontal gene transfer in bacteria can occur through mechanisms such as conjugation, transduction and transformation, which facilitate the passage of DNA across the cell wall. Here, Kapteijn et al. show that cell wall-deficient bacteria can take up DNA and other extracellular materials via an endocytosis-like process.

## Introduction

Bacteria are constantly exposed to changing environmental conditions and rely on their cell envelope for protection. The cell envelope consists of a cell membrane and a cell wall to separate the internal from the external environment. The cell membrane is a phospholipid bilayer that encloses the cytoplasm and functions as a selective barrier. The cell wall consists of thick peptidoglycan (PG) layer for Gram-positive bacteria and a thinner PG layer surrounded by an outer membrane for Gram-negative bacteria. The peptidoglycan layer is an important mesh-like structure that not only provides protection against mechanical stress and turgor pressure, but also defines cell shape and rigidity.

To facilitate the selective passage of macromolecules across the cell envelope, bacteria have evolved specialized and sophisticated transport systems^1^. For instance, naturally transformable bacteria rely on protein complexes for DNA uptake, with components similar to type IV pili or type II secretion systems. Active transport of DNA across the cell wall is facilitated by the retraction of pilus structures that bind DNA^2,3^. DNA-binding and pore-forming proteins are then used to translocate the DNA across the cell membrane.

Although the cell wall is a vital structure for most bacteria, some bacteria naturally lack a cell wall, or can shed their wall under specific conditions. Examples include the members of the Mollicutes, that are parasitic and live in specific osmotically protective environments such as human mucosal surfaces or the phloem sieve tubes of plants^4^. Prolonged exposure to environmental stressors such as cell wall-targeting agents generates so-called L-forms, which are cells that can proliferate without their cell walls. Reproduction of L-forms is independent of the canonical FtsZ-based division machinery^5^ and is driven by an imbalance in the surface area to volume ratio of cells caused by the upregulation of membrane synthesis leading to spontaneous blebbing, tabulation, and vesiculation^6,7^. These primitive cell-like characteristics make L-forms an attractive model system to study the evolution of early life^8,9^.

**Fig. 1:**
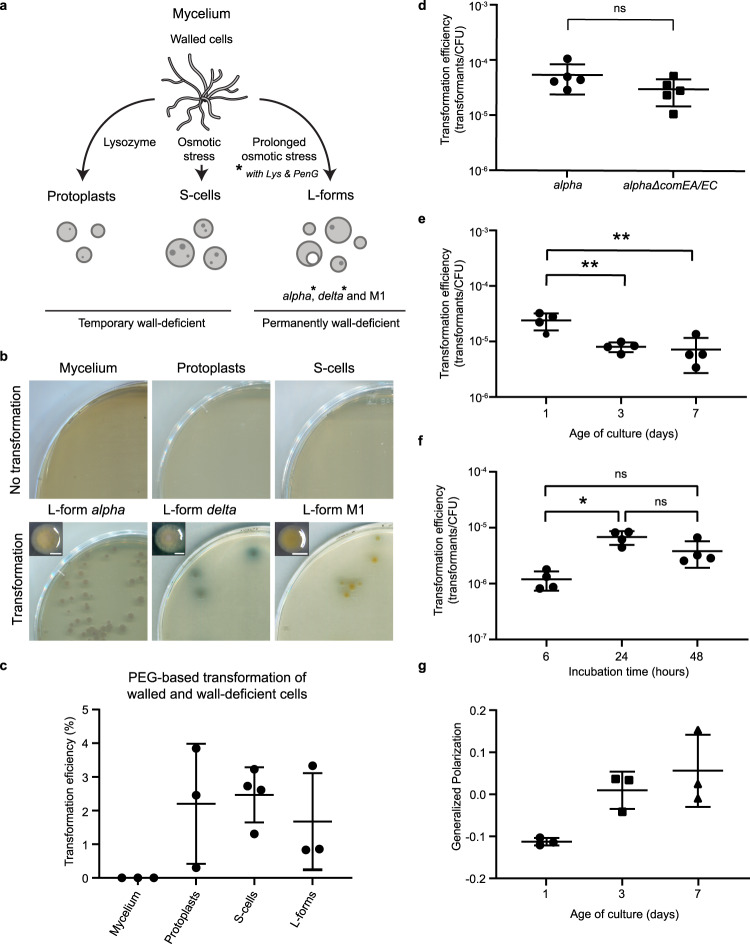
DNA uptake by cell wall-deficient cells is independent of homologs of the competence proteins ComEA and ComEC and correlates with membrane fluidity. **a** Schematic representation of the generation of cell wall-deficient cells of *K. viridifaciens*. Temporary wall-deficient cells include protoplasts, obtained from mycelial cells by the action of lysozyme, and S-cells, extruded from the hyphal tips in a medium with high osmotic pressure. Permanently wall-deficient L-forms are generated after prolonged incubation of mycelium under a high osmotic pressure (line M1), with the optional supplementation of lysozyme (Lys) and penicillin G (PenG) (lines *alpha* and *delta*). **b** Mycelium (*n* = 3), protoplasts (*n* = 5 from two experiments), S-cells (*n* = 7 from two experiments) and L-form lines *alpha* (*n* = 3 for both 4-and 6-day-old cells), *delta* (*n* = 2 for both 3-and 7-day-old cells) and M1 (*n* = 1 for 3-day-old cells) were incubated with plasmid DNA (pRed*) for 18–24 h, plated on selective medium and incubated at 30 °C to select for transformed cells. A close-up of the colony morphology of the L-forms is given. Scale bars indicate 1 mm. Note that only L-forms show consistent DNA uptake. **c** Polyethylene glycol (PEG)-based transformation efficiency of *K. viridifaciens* mycelium, protoplasts, S-cells, and L-forms using plasmid DNA (pRed*) as given in the percentage of transformed colonies. *n* = 3 biological replicates except for S-cells (*n* = 4). **d** Transformation efficiency of 7-day-old *alpha* and *alpha*Δ*comEA/EC* after 24-h incubation with pFL-*ssgB*. CFU = colony forming units. ns = not significant (*n* = 5 biological replicates, two-sided independent t-test, *t*(8)=1.572, *P* = 0.155). **e** Transformation efficiency of 1-, 3-, and 7-day-old *alpha* after 24 h incubation with pFL-*ssgB*. Asterisks (**) indicate *P* ≤ 0.01 (*n* = 4 biological replicates, one-way ANOVA, F (2, 9) = 12.16, Tukey post hoc test, *P* = 0.006 (1–3 day) and 0.005 (1–7 day)). **f** Transformation efficiency of 7-day-old *alpha* incubated with pRed* for 6, 24, or 48 h. Asterisk (*) indicates a statistically significant difference between 6- and 24-h incubation (two-sided Kruskal–Wallis test, H(2) = 8.769, *P* = 0.012 with Dunn’s pairwise test, including Bonferroni correction, gives *P* = 0.010 for 6 and 24 h, *P* = 0.233 for 6 and 48 h and *P* = 0.718 for 24 and 48 h). ns not significant. *n* = 4 biological replicates. **g** Generalized polarization (GP) as a measurement of membrane fluidity of 1-, 3-, and 7-day-old *alpha* using the membrane dye Laurdan. Lower GP indicates a higher membrane fluidity. *n* = 3 biological replicates. Data in (**c**–**g**) are represented as mean ± SD with individual data points. Source data are provided as a Source Data file.

Some filamentous actinomycetes, such as the mycelium-forming *Kitasatospora viridifaciens*, have the ability to transiently shed their cell wall under conditions of hyperosmotic stress (Fig. 1a)^7^. Contrary to L-forms, S-cells are not able to proliferate without their cell wall, although these cells are able to revert to the mycelial mode-of-growth after rebuilding their cell wall. Temporary cell wall-deficient cells can also be generated artificially from walled bacteria via enzymatic removal of the cell wall, for example, by the action of lysozyme that degrades peptidoglycan. This leads to the formation of protoplasts or spheroplasts, which are widely used for genetic engineering purposes, often involving the use of polyethylene glycol (PEG) to allow DNA entry into the cell^10^. Although this PEG-based transformation is a widely used technique for the transformation of filamentous actinomycetes, it has never been unambiguously shown whether *K. viridifaciens* walled cells, or its natural cell wall-deficient cells, are capable of natural genetic transformation without using PEG. How the absence of the cell wall affects the natural uptake of macromolecules such as DNA from the environment is unknown.

In this work we show that L-forms of the filamentous actinomycete *K. viridifaciens* can take up DNA independent of the canonical natural genetic transformation machinery. Instead, uptake is facilitated by a mechanism of horizontal gene transfer that involves the invagination of the cell membrane leading to internal vesicle formation. Furthermore, we show that this mechanism is robust and allows the non-specific uptake of other macromolecules from the environment as well. Given that L-forms are considered a model for early cellular life, our work provides insight into how such ancient cells may have acquired large biomolecules and nanoparticles from the environment without the need for complex transport machineries.

## Results

### DNA uptake by wall-deficient cells

It is unknown whether walled or wall-less cells of *K. viridifaciens* are capable of natural genetic transformation. To analyse this, walled mycelial cells, temporary wall-less S-cells and protoplasts, and permanently wall-less L-forms (*alpha*) were freshly harvested and resuspended in an osmotically-stable LPB medium. Subsequently, cells were incubated for 18–24 h with plasmid DNA (pRed*) containing an antibiotic resistance cassette and plated on selective and nonselective media to allow the detection of transformed cells. Notably, L-forms were consistently able to take up DNA, unlike mycelium, protoplasts, and S-cells (Fig. 1b). DNA uptake was not restricted to one L-form line, but was observed with distinct L-form cell lines obtained from walled cells of *K. viridifaciens* grown in LPB medium with (lines *alpha* and *delta*) or without (line M1) penicillin and lysozyme^7,11^. No transformants were obtained with *alpha* when intact or fragmented genomic DNA was used, or when using methylated plasmid (Supplementary Fig. 1a, b). While natural genetic transformation was restricted to L-forms, all wall-deficient cells could be chemically transformed using polyethylene glycol (PEG), with protoplasts, S-cells, and L-forms having an average transformation efficiency between 1.7–2.5% (Fig. 1c). The addition of PEG also enabled the transformation of *alpha* with genomic DNA, even if this was present in a crude cell extract (Supplementary Fig. 1c). On the other hand, use of methylated DNA prevented chemical transformation, indicating that PEG-based transformation is possible with different types of DNA, but is limited by the presence of a different methylation pattern. By contrast, walled cells could not be transformed either with or without PEG (Fig. 1b, c). These results show that in these conditions, L-forms can naturally take up DNA, unlike walled cells, S-cells and protoplasts.

### The canonical transformation machinery is not required for DNA uptake

Naturally transformable bacteria use a specialized DNA translocation machinery with similarities to type IV pili or type II secretion systems to take up external DNA^2^. Similar components of this canonical system might also be involved in DNA uptake by L-forms. A BlastP search using the DNA-binding protein ComEA and channel protein ComEC of the naturally transformable bacterium *Bacillus subtilis* str. 168 against *K. viridifaciens* yielded two significant hits: BOQ63_029625 (helix-hairpin-helix domain-containing protein) and BOQ63_029630 (ComEC/Rec2 family competence protein), respectively (Supplementary Table 1 and Supplementary Fig. 1d). The *B. subtilis* helicase/DNA translocase ComFA resulted in a hit to a putative Mfd-encoding gene (BOQ63_020315), a widely conserved bacterial protein that mediates transcription-coupled DNA repair^12^. No other orthologues were found that correlated to proteins involved in DNA transport across the cell envelope for *B. subtilis*, the Gram-negative *Neisseria gonorrhoeae*^13^, or for the T4SS-related DNA uptake system of *Helicobacter pylori*^14^ (Supplementary Table 1). L-forms lack an intact peptidoglycan-based cell wall, and therefore, DNA must only cross the cell membrane for internalization. In naturally transformable bacteria, ComEA and ComEC function in DNA transport across the cell membrane^13,15,16^. Although the role of the putative *comEC* and *comEA* genes in *K. viridifaciens* is unknown, as its walled cells did not show DNA uptake, we wondered whether these proteins could be involved in DNA uptake in L-forms. Therefore, we replaced the putative *comEC* and *comEA* genes in the L-form strain *alpha* by an apramycin resistance cassette (Supplementary Fig. 1d, e). Strikingly, the simultaneous deletion of the *comEA* and *comEC* genes did not affect the transformation efficiency (two-sided independent *t*-test, *t*(8) = 1.572, *P* = 0.155), indicating that DNA uptake by L-forms occurs independently of genes homologous to this canonical DNA translocation machinery (Fig. 1d).

### High membrane fluidity is not sufficient for DNA uptake

The ability to take up DNA for natural transformation is regulated differently amongst naturally transformable bacteria and can be constitutively active or restricted to a specific growth phase, as reviewed by Blokesch (2016)^17^. One of the factors controlling the development of competence for DNA uptake in *B. subtilis* is the growth phase^18,19^. To study if culture age is also affecting the DNA uptake ability of L-forms, cells from differently aged cultures were subjected to a transformation assay. Cells from 1-day-old cultures of *alpha* take up DNA more easily than from 3- or 7-day-old cultures (one-way ANOVA, *F* (2,9) = 12.16, Tukey post hoc test, *P* = 0.006 and 0.005 respectively) (Fig. 1e). To test if shorter or longer incubation times affect DNA uptake, the transformation efficiency of 7-day-old *alpha* was determined after 6-, 24-, and 48-h incubation with DNA (Fig. 1f). Transformation was detected after 6-h incubation and increased after 24 h (two-sided Kruskal–Wallis test, H(2) = 8.769, *P* = 0.012 and Dunn’s pairwise test with Bonferroni correction gives *P* = 0.010 for 6 and 24 h, *P* = 0.233 for 6 and 48 h, and *P* = 0.718 for 24 and 48 h). It is not unlikely that differences in membrane properties that occur during cellular growth may in turn affect the DNA uptake ability. Membrane fluidity is a measure of the average viscosity of the lipid bilayer, which can affect the positioning and movement of proteins and lipids within the membrane^20^. Higher membrane fluidity is characterized by increased fatty acid disorder, lower lipid packing and higher diffusion rates, which can lead to increased membrane permeabilization^21,22^. Analysis of the membrane fluidity of the differently aged cultures indicated that the increased DNA uptake ability may correlate positively with the fluidity of the membrane, as deduced from the generalized polarization (GP, a lower GP indicating higher fluidity)^23^ (Fig. 1g), although no statistically significant differences were observed (Welch one-way ANOVA, *F*(2, 2.798) = 13.226, *P* = 0.038, with Games-Howell post hoc test: 1–3 day *P* = 0.068; 1–7 day *P* = 0.134; 3–7 day *P* = 0.711, *n* = 3). A relatively low fluidity might explain why temporary wall-deficient protoplasts and S-cells cannot take up DNA naturally. However, the fluidity of protoplasts was within the range of 1- to 7-day-old cultures as measured using a plate assay (Supplementary Fig. 2a). Subsequent analysis of the GP by fluorescence microscopy imaging showed that although protoplasts and S-cells tend to have less fluid membranes, these values stay within the range of the membrane fluidity of 1- to 7-day-old L-forms (Supplementary Fig. 2b). Therefore, although membrane fluidity may contribute to efficient DNA uptake, it is not sufficient to explain this process.

### Internal vesicle formation in L-forms results in DNA uptake

**Fig. 2:**
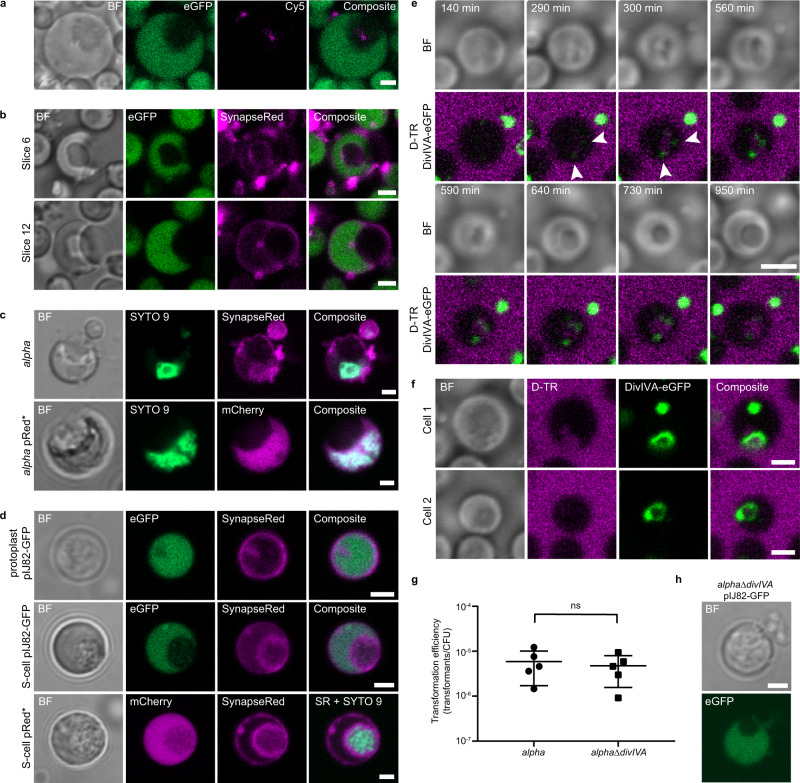
Formation of internal vesicles and uptake of extracellular material in L-forms. **a** Representative fluorescence micrograph of *alpha* pIJ82-GFP (cytoplasmic eGFP; green) incubated with Cy5-labeled plasmid DNA (pFL-*ssgB*; magenta) for 3 days (*n* = 3 observations from one experiment). BF brightfield. See also Supplementary Movie 1. **b** Incubation of *alpha* pIJ82-GFP after overnight incubation with the membrane-impermeable dye SynapseRed C2M (SynapseRed; magenta), showing two z-slices at different heights of one L-form cell. A representative micrograph of six observations from one experiment is shown. **c** Representative micrograph of *alpha* (*n* = 7 observations from one incubation) and *alpha* pRed* (*n* = 9 observations from one incubation) stained with SYTO 9 (green) to indicate chromosomal DNA. *alpha* is stained with SynapseRed C2M (SynapseRed; magenta) to visualize cell membranes, whereas (absence of) cytoplasmic mCherry for *alpha* pRed* (magenta) indicates the presence of an internal vesicle. Cells were imaged directly after the addition of the fluorescent dyes. **d** Representative images of protoplasts and S-cells of *K. viridifaciens* pIJ82-GFP producing cytoplasmic eGFP incubated with SynapseRed (SR) for 72 h (top rows, at least six observations from two independent experiments with S-cells and protoplasts), and S-cells of *K. viridifaciens* pRed* producing cytoplasmic mCherry incubated with SynapseRed and SYTO 9 for 72 h (bottom row, three observations from one experiment). Note that the presence of internal membrane structures and/or DNA can cause a reduction in cytoplasmic fluorescence emission. **e** Stills of a 950-min timelapse imaging experiment of *alpha* producing DivIVA-eGFP (*alpha* pKR2) (green) incubated with 3 kDa Dextran-Texas Red (D-TR; magenta) (*n* = 1). Arrows indicate localization of DivIVA-eGFP. See also Supplementary Movie 2. **f** Representative micrograph of the formation of foci and ring-structures of DivIVA-eGFP in *alpha* pKR2 (green) after overnight incubation with D-TR (magenta) (more than 50 observations from one experiment). Note that L-forms are able to take up fluorescently stained DNA and dextran by the formation of internal vesicles. **g** Transformation efficiency of 7-day-old *alpha* and *alpha*Δ*divIVA* using pFL-*ssgB*. ns not significant (two-sided independent *t*-test, *t*(8) = 0.489, *P* = 0.638). CFU colony forming units. Data were represented as mean ± SD with individual data points, *n* = 5 biological replicates. **h** L-forms without DivIVA can produce internal vesicles as shown for 5-day-old *alpha*Δ*divIVA* pIJ82-GFP producing cytoplasmic eGFP (*n* = 2 observations from one culture). Scale bars indicate 2 μm. Source data are provided as a Source Data file.

To further investigate the mechanism facilitating DNA uptake by L-forms, we added Cy5-labeled plasmid DNA to L-forms expressing cytoplasmic eGFP. After 3 days of incubation, labeled plasmid DNA was found either on the outside of the L-form cell membrane, or within an apparent internal vesicle (Fig. 2a, Supplementary Movie 1, and control Supplementary Fig. 3a). As these internal vesicles were devoid of eGFP, we reasoned that they could have originated by an invagination process of the membrane, whereby extracellular material becomes trapped inside the vesicles. To test this directly, we incubated eGFP-expressing L-forms with the fluorescent dye SynapseRed C2M (SynapseRed). Given that styryl dyes, such as SynapseRed (equivalent to FM5-95), cannot diffuse through cell membranes^24,25^, any fluorescent signal on the membranes surrounding internal vesicles would be a strong argument that such vesicles were derived from the cell membrane. Indeed, SynapseRed was found to not only stain the cell membrane of the L-forms but also the membranes of internal vesicles after overnight incubation (Fig. 2b). Staining with SYTO 9 further indicated that chromosomal DNA was present in the cytoplasm but not inside internal vesicles (Fig. 2c). Incubation of protoplasts producing cytoplasmic eGFP with SynapseRed showed that areas with less cytoplasmic eGFP fluorescence were caused by the presence of internal membrane structures rather than by formation of internal vesicles (Fig. 2d). Similar incubation of S-cells showed the presence of internal vesicle-like structures devoid of cytoplasmic eGFP. However, unlike for L-forms, subsequent staining of S-cells that produce cytoplasmic mCherry with SYTO 9 indicated that these dark regions were filled with chromosomal DNA. As SynapseRed is a membrane-impermeable dye, the staining of membrane structures in S-cells may reflect enhanced membrane permeability instead of staining due to the invagination of the cell membrane. To test the diffusion of the dye across the cell membrane, L-forms and S-cells were incubated with SynapseRed at 30 °C and at 4 °C to enable and prevent possible membrane invagination, respectively. Whereas internal membrane was stained in S-cells both at 30 °C and 4 °C, this was only observed at 30 °C for L-forms (Supplementary Fig. 3b). This suggests that the staining of a membrane in S-cells by SynapseRed is due to permeability of the cell membrane for the dye, in contrast to L-forms, in which staining is a result of invagination of the cell membrane.

To compare the presence of putative internal vesicle structures in the different cell types, the percentage of L-forms, protoplasts and S-cells with these structures was quantified. Cells producing cytoplasmic mCherry (*alpha* pRed* or S-cells and protoplasts derived from *K. viridifaciens* pRed*) were incubated with SynapseRed for 0 and 3 days to stain membranes. DNA was visualized with SYTO 9 before imaging. The number of cells with regions lacking fluorescence emission from the cytoplasm, DNA, and membrane, which could indicate the presence of internal vesicles, was quantified (Supplementary Table 2). Using this method, L-forms have around 6- to 11-fold higher occurrence of such regions than S-cells (L-forms: 24.5 and 14.7%; S-cells: 2.2 and 2.6% after 0 and 3-day incubation, respectively), and putative vesicles were rarely observed for protoplasts (<0.5%). If these regions in S-cells and protoplasts would be actual internal vesicles, their occurrence is likely not sufficient to detect consistent transformation with DNA. Taken together, these results strongly suggest that the observed vesicles inside L-forms originate from the invagination of the cell membrane, whereby extracellular DNA may become trapped inside such vesicles, whereas this is not evident for S-cells and protoplasts.

### L-forms take up extracellular material via an endocytosis-like mechanism

In eukaryotes, endocytosis is a process that enables the uptake of external cargo via internal vesicle formation, which is eventually degraded or recycled^26,27^. Fluorescently labeled dextrans are widely used as markers for endocytosis in eukaryotes as they cannot pass the cell membrane^28,29^. To identify if such an endocytosis-like process could be present in L-forms and to visualize the uptake of external materials, we incubated the cells with Dextran-Texas Red (D-TR) and performed timelapse imaging. The L-form strain used also expresses DivIVA-eGFP, which has a strong affinity for negatively curved membrane regions (*alpha* pKR2)^30^. Such regions are expected to be formed upon the invagination of the membrane. After 290 min of incubation, D-TR was visible inside the L-form and faint spots of DivIVA-eGFP started to appear adjacent to this region (Fig. 2e and Supplementary Movie 2). This progressed to a clear inward bulging of the cell membrane with two foci of DivIVA-eGFP on either side of the invaginated membrane and an inflow of D-TR (*t* = 560 min). After 640 min an internal vesicle was formed that contained D-TR. In other cells, DivIVA-eGFP appeared to form a ring-like structure, which sometimes enveloped the invaginating membrane (Fig. 2f cell 1 and 2). The presence of DivIVA near the site of invagination implies the presence of negatively curved regions in the membrane. Notably, DivIVA is not required for vesicle formation or DNA uptake, as the deletion of *divIVA* (*alpha*Δ*divIVA*) had no effect on transformation (two-sided independent *t*-test, *t*(8) = 0.489, *P* = 0.638) (Fig. 2g), and internal vesicles were still formed by this strain (Fig. 2h). Furthermore, internalization of D-TR was also observed in L-forms that did not express DivIVA-eGFP, indicating that uptake is not a consequence of the presence of the fusion protein (Supplementary Fig. 3c). Incubation of protoplasts and S-cells with D-TR up to 72 h did not result in clear D-TR encapsulation in internal vesicles (Supplementary Fig. 3d). To quantify this, cells producing cytoplasmic eGFP were incubated with D-TR or PBS for 72 h. The percentage of cells with regions lacking eGFP but containing D-TR in this region was counted. Repeated incubation (in duplo) resulted in uptake of D-TR in around 6% of L-form cells and no uptake for the control with PBS (Supplementary Table 3). No clear uptake was observed for S-cells and protoplasts incubated with D-TR when compared to control cells incubated with PBS (S-cells: 0.9 and 1.7% with D-TR versus 0 and 2.1% with PBS; protoplasts: 0 and 1.1% with D-TR versus 0 and 0.8% with PBS). Altogether, these results show that the invagination of the cell membrane of L-forms can lead to internal vesicle formation and may represent an endocytosis-like mechanism allowing uptake of molecules, including DNA, from the environment.

### Lipid nanoparticles are internalized via vesicle formation in an energy-dependent manner

**Fig. 3:**
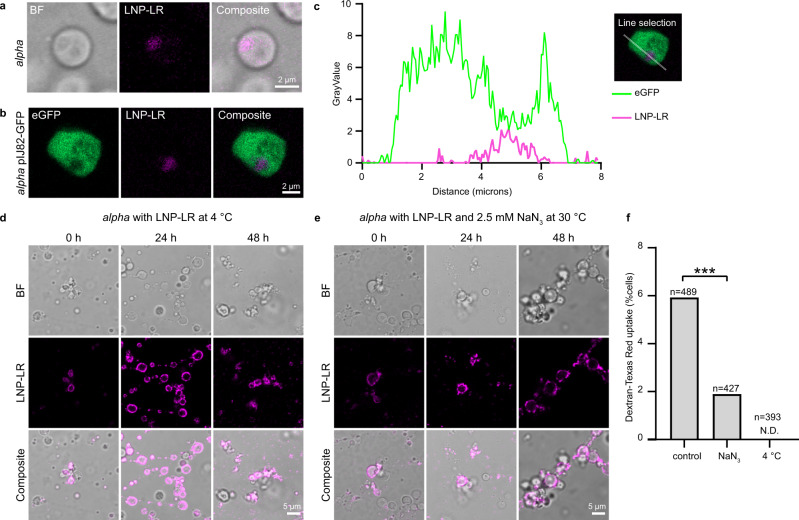
Localization of lipid nanoparticles in internal L-form vesicles. **a**, **b** Representative micrographs of localization of LNP-LR (Lipid nanoparticles containing 18:1 Liss Rhod PE; magenta) in internal vesicles of *alpha* (a; *n* = 5 observations) and *alpha* pIJ82-GFP (b; *n* = 2 observations) after an overnight and 3-day incubation experiment at 30 °C, respectively. **c** Density profile plot of gray values (pixel intensity) of corresponding line selection of *alpha* pIJ82-GFP (**b**) incubated with LNP-LR showing that a decrease in cytoplasmic eGFP emission correlates with an increase in LNP-LR emission. **d**, **e** Localization of LNP-LR during incubation with *alpha* at 4 °C (**d**) or in the presence of 2.5 mM sodium azide (NaN_3_) at 30 °C (**e**) after 0-, 24-, and 48-h incubation. Similar results were obtained with 1 and 10 mM sodium azide (See Supplementary Fig. 4) Images were obtained from one experiment. **f** Percentage of *alpha* pIJ82-GFP cells showing Dextran-Texas Red (D-TR) uptake after 3-day incubation at 30 °C (control), in the presence of 2.5 mM sodium azide (NaN_3_) or incubated at 4 °C. A significant reduction in D-TR uptake was observed for sodium azide (two-proportion *z*-test, *z* = 3.111, one-sided, *P* = 0.00093) and after incubation at 4 °C no uptake was detected (ND). Asterisks (***) indicate *P* ≤ 0.001. The percentage of cells with D-TR uptake and the total number of cells analysed is given for each condition and is based on combined cell counts from two replicate incubations. Note that incubation of L-forms with lipid nanoparticles (average size of 150 nm) results in their localization inside internal vesicles, and that uptake of external particles is inhibited by incubation at 4 °C or with sodium azide. Source data are provided as a Source Data file.

Lipid nanoparticles (LNPs) are non-viral particles that are used to deliver nucleic acids and drugs to human cells via endocytosis^31^. LNPs do not have a lipid bilayer structure, but consist of an electron-dense, hydrophobic core of lipids that encapsulate nucleic acids by electrostatic interactions and are surrounded by a layer of PEG-lipids^31^. Internalized LNPs are located in endosomes that are membrane-bound organelles of the endocytic pathway. Subsequent acidification causes the ionizable lipids of the LNPs to become positively charged, which allows the LNP to destabilize the endosomal membrane and deliver its cargo into the cell. LNPs can also be fluorescently tagged by the incorporation of fluorophore-conjugated phospholipids. To further explore the ability of L-forms to take up large external particles, the cells were incubated with rhodamine-labeled LNPs (LNP-LR, containing 18:1 Liss Rhod PE) with an average size of 150 nm, to allow their detection inside L-forms. After addition of LNP-LR to 7-day-old L-forms, a clear fluorescent signal, likely generated by multiple LNP particles, could be detected inside cells after overnight incubation, as well as localization of LNPs to the cell membrane (Fig. 3a and Supplementary Fig. 4a, b). When L-forms were used that produced eGFP in the cytoplasm, vesicles only contained LNPs and not eGFP, strongly suggesting that the LNPs had been internalized in vesicles devoid of the cytoplasm (Fig. 3b, c and imaging control Supplementary Fig. 4c). Importantly, the addition of the metabolic inhibitor sodium azide (1, 2.5, or 10 mM), which targets the respiratory chain^32^, or incubation of cells at 4 °C affected the localization of LNP-LR (Fig. 3d, e and Supplementary Fig. 4d–i). These conditions are commonly used to inhibit endocytosis by repressing energy production^33,34^. Under such conditions, the LNPs appeared to localize to the cell membrane rather than inside the cell. The inhibiting effect of sodium azide and incubation at 4 °C on uptake of extracellular material by L-forms was quantified using D-TR, as capturing the uptake of LNP-LR was too infrequent to quantify accurately. Strikingly, a significant reduction of D-TR uptake by L-forms was observed in the presence of 2.5 mM sodium azide (5.9% versus 1.9% of cells showing D-TR uptake for control and sodium azide, respectively (two-proportion *z*-test, *z* = 3.111, one-sided *P* < 0.001), and incubation of L-forms at 4 °C completely inhibited D-TR uptake (Fig. 3f). These results are consistent with an uptake process in L-forms that is energy-dependent, whereby external material is internalized by a membrane invagination process.

### High-resolution imaging of L-forms using cryo-FIB-SEM

**Fig. 4:**
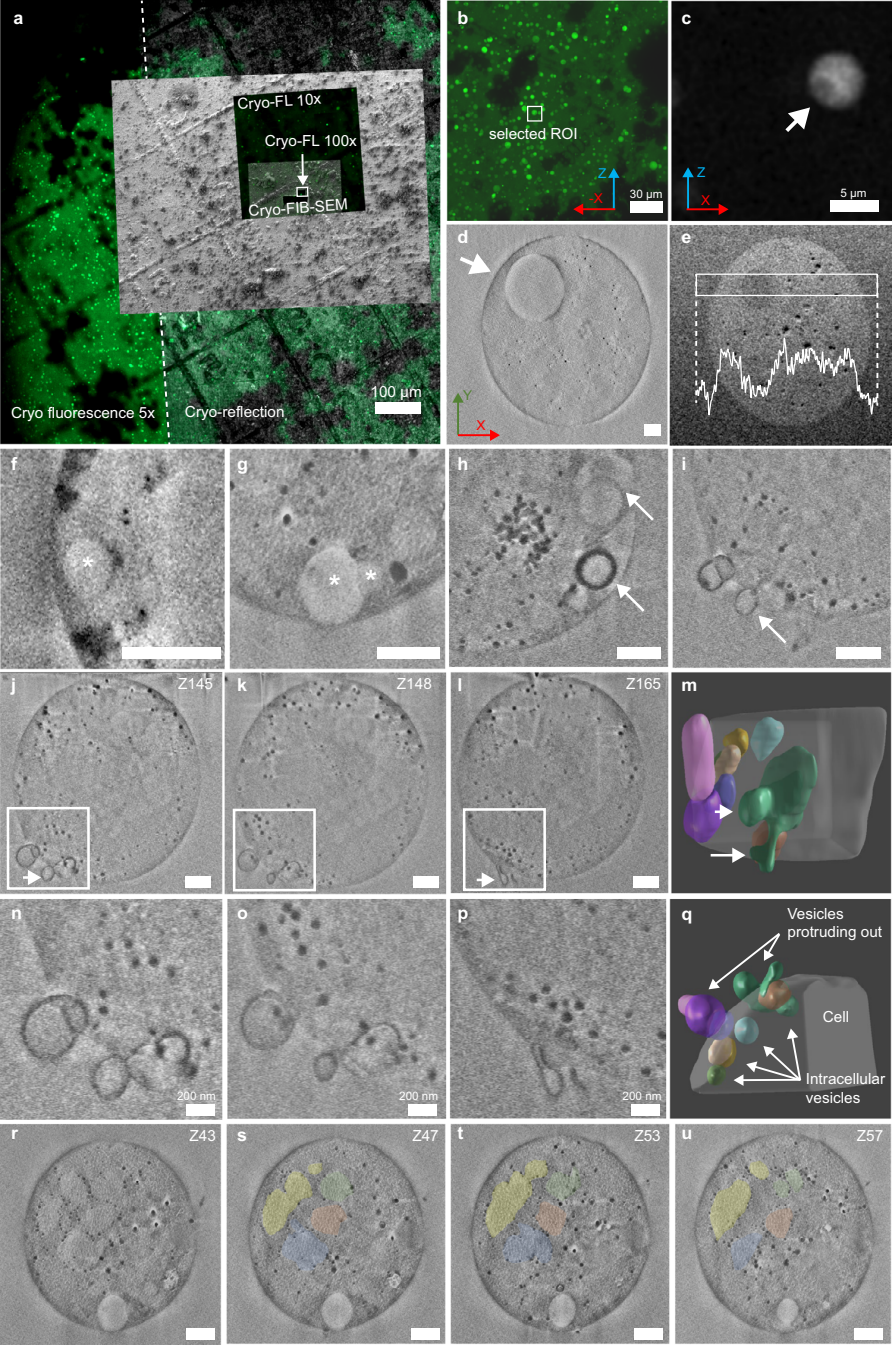
3D cryo-fluorescence and cryo-FIB-SEM of L-forms reveal their ultrastructure in high resolution. **a** Example of correlated fluorescence and electron micrographs of *alpha* pIJ82-GFP (Zen Connect image) as performed with all imaged cells. A finderTOP raster visible both in fluorescence and electron microscopy facilitates alignment between the two imaging modules. Squares indicate different regions of interest, imaged at higher resolution. FIB-SEM focused ion beam— scanning electron microscopy, FL fluorescence light. **b** Example of a higher resolution image of a selected region of interest (ROI), showing many fluorescent cells. **c** Fluorescence micrograph of the L-form depicted by the white box in (**b**), showing intracellular dark sphere (~1 μm, white arrow), as was performed to select all cells of interest. The X, Y, and Z arrows in (**b**–**d**) indicate the 3D orientation of the imaged cell as observed in 3D FIB-SEM. **d** Scanning electron microscopy (SEM) image (SE, Inlens) of cell in (**c**) (size ~6 μm) with an arrow indicating the internal vesicle (*n* = 1 cell). **e** Superposition of five consecutive slices (backscattered images) of cell in (**d**). Inset: Density profile plot (white) of the average gray values (pixel intensity) for the region in the white box (*n* = 1 cell). **f**–**i** FIB-SEM slices showing different types of internal vesicles from three imaged cells. Vesicles lining the cell membrane of cells of around 3.5 μm in size (**f**, **g**). Asterisks indicate vesicles in (**f**, **g**). Vesicle complex (**h**) with different membrane thickness of vesicles indicated with white arrows. See also Supplementary Fig. 7a and Supplementary Movie 3. (i) Membrane protrusions as indicated with a white arrow. **j**–**q** Analysis of the interconnected vesicles of the cell in (i) (*n* = 1). **j**–**l** Three consecutive slices showing the interaction of different vesicles. **n**–**p** show higher magnification of the regions in white boxes in **j**–**l**, respectively. **m**, **q** 3D segmentation of **n**–**p**. While some of the vesicles are intracellular, others protrude out of the cell. A complete connected vesicle structure is shown in green (**m**, **q**) and indicated by white arrows (**i**, **j**, **l**, **m**). See Supplementary Fig. 7b–d and Supplementary Movie 4. The cell in panels **h**–**q** is around 4 μm in size. **r**–**u** Regions with different contrast (indicated by colored regions) are lined with black particles representing putative lipid bodies as shown for one cell (similar regions observed in three cells). This cell is ~3.6 μm in size and relates to panel (**g**). The size distribution of the black particles is 25 to 60 nm. Scale bars represent 500 nm unless otherwise specified. Source data are provided as a Source Data file.

To better understand the ultrastructure and composition of intracellular vesicles, L-forms were imaged using 3D cryo-correlative light and electron microscopy (cryo-CLEM) (Fig. 4a). Cryo-FIB-SEM (focused ion beam—scanning electron microscopy) allows the 3D high-resolution imaging of L-forms and internal vesicles. The cryogenic sample preparation and imaging ensure that the L-forms are visualized in a near-to-native state via rapid freezing under high-pressure conditions that allow the transformation of liquid to amorphous ice^35–37^.

Following high-pressure freezing, cells with putative intracellular vesicles were detected based on internal darker regions lacking cytoplasmic eGFP using *alpha* pIJ82-GFP (Supplementary Fig. 5). Specific L-forms (example of selection in Fig. 4b, c) were imaged in detail using cryo-FIB-SEM. The reduction in cytoplasmic eGFP indeed matched the presence of internal vesicles as detected by FIB-SEM (Fig. 4c, d, white arrow), in line with previous results (Fig. 2b). In addition, the composition of the cytoplasm and internal vesicle content was different, as measured using the InLens energy selective backscattered (EsB) detector which provides contrast based on the distribution of heavier elements (Fig. 4e). Analysis of the pixel intensity indicated that the contrast level inside the internal vesicle was similar to the extracellular environment, whereas the cytoplasm had a higher contrast. Moreover, an over-exposure experiment showed that the vesicle has the same capacity to absorb the electron dose as the medium outside, different from the rest of the cell (Supplementary Fig. 6a, b). These results support the finding that internal vesicles contain extracellular medium and are formed via membrane invagination (Fig. 2).

Further high-resolution imaging indicated the presence of multiple internal vesicles within individual cells (Fig. 4f–i, Supplementary Fig. 6c–e). Most detected vesicles were lining the cell membrane (Fig. 4g and Supplementary Fig. 6c–e), varied in size and membrane thickness (Fig. 4h) and could even be present inside larger vesicles (Fig. 4h and Supplementary Fig. 7a). The presence of vesicles within other vesicles has also been observed using fluorescence microscopy (Supplementary Fig. 3c, white arrow), showing a non-fluorescent vesicle within a larger vesicle encapsulating D-TR. In addition, vesicles could be observed budding out of the cell membrane (Fig. 4i). 3D reconstruction of the budding vesicles based on contour tracing revealed that these were either an extension of an internal vesicle, or remained connected to internal vesicles, forming a complex (Fig. 4j–q, Supplementary Fig. 7a–d, and Supplementary Movies 3, 4).

In some cases, cells contained intracellular regions with different gray values from the rest of the cell (Fig. 4r–u). These regions had a size distribution of 300 to 800 nm, did not line the cell membrane, and were surrounded by dark particles of around 25–60 nm in diameter. It could be possible that these dark particles are lipid bodies, compared to previous cryo-FIB-SEM observations^38,39^. A potential interpretation is that the internal regions are vesicles of which the enclosing lipid membrane has partially degraded. The lipids and lipidic degradation products may have accumulated in lipid droplets that result in the observed black particles. To capture internal vesicle degradation, overnight timelapse imaging was performed on L-forms expressing cytoplasmic mCherry (*alpha* pRed*). Vesicles were identified by lack of cytoplasmic mCherry, and cells were imaged at different Z-heights to confirm that the vesicle had not simply moved location. Vesicle disruption was observed using 2-day-old L-forms resuspended in fresh LPB medium (Supplementary Movie 5). Two more events of vesicle disruption were captured after performing a successive timelapse on the same sample, but with different cells (Supplementary Movies 6, 7). All imaged vesicles were already present in the cells at the start of the timelapse. Whereas the time elapsed until vesicle disruption varied greatly (after 1 h or after 13 and 17 h for the second experiment), the disruption process itself occurred within 15 min, which was the time interval between consecutive images. These findings strengthen the model that internal vesicles of L-forms can disrupt and, in this way, release their contents in the cytoplasm.

**Fig. 5:**
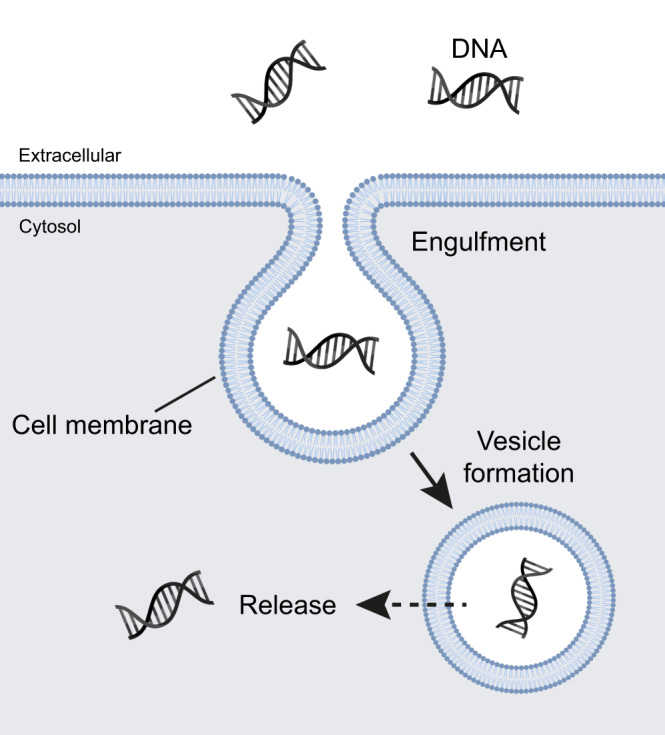
Proposed model for DNA uptake by internal vesicle formation in L-forms. Invagination of the cell membrane leads to the formation of internal vesicles in L-forms. As the cell membrane bulges inward, extracellular liquid containing DNA or other macromolecules is engulfed. The mechanism underlying the invagination process is energy-dependent and could be based on increased membrane synthesis and high membrane fluidity or mediated by proteins similar to those involved in eukaryotic endocytosis, such as cytoskeletal proteins. DNA is released from internal vesicles by an unknown process (indicated by a dashed arrow), which may involve vesicle disruption. Image created with BioRender.com.

These results further confirm that the internal vesicles observed in *K. viridifaciens* L-forms contain external medium and can be formed by the invagination of the cell membrane. L-forms can contain multiple vesicles of varying sizes, in some cases forming clusters or complexes of vesicles that can protrude out of the cell membrane. Internal vesicles may release their contents into the cell after vesicle degradation. These findings support a model for the uptake of macromolecules such as DNA by engulfment, followed by the release of the cargo after vesicle disruption (Fig. 5).

## Discussion

The bacterial cell wall is an important protective barrier to the environment, providing stress resistance and enabling the selective passage of molecules. However, in recent years it has become clear that under some conditions, bacteria may also thrive without this layer. Prolonged exposure to environmental stresses, such as cell wall-targeting agents or a high osmotic pressure, can induce the formation of L-forms that efficiently proliferate without their cell wall^7^. The consequences of such a wall-deficient bacterial lifestyle on their ability to take up DNA are largely unknown. Here we provide evidence that L-forms may take up DNA and other macromolecules via engulfment and the subsequent formation of internal vesicles (Fig. 5).

Well-known mechanisms for HGT are natural transformation, transduction, and conjugation^40^. These mechanisms require sophisticated machinery to enable transport of DNA across the cell envelope. We here show that wall-deficient cells such as protoplasts, S-cells, and L-forms of *K. viridifaciens* take up DNA using PEG. Importantly, L-forms are the only wall-deficient cells that achieve consistent spontaneous transformation using plasmid DNA without PEG. No transformation was observed when using genomic DNA without using PEG. This is likely due to the ~200-fold lower number of gDNA molecules used as compared to plasmid DNA, as well as the requirement for double recombination of genomic DNA with the chromosome for stable integration of the antibiotic resistance cassette, which likely leads to a lower transformation efficiency.

Naturally transformable bacteria use a canonical and complex system for DNA uptake across the cell wall and cell membrane. The latter step requires the DNA-binding protein ComEA and the pore-forming channel protein ComEC, with homologs found across naturally transformable Gram-positive and Gram-negative species (e.g., ComE and ComA in *N. gonorrhoeae*). Disruption of either of these proteins typically results in a drastic reduction or even absence of transformation^15,16,41,42^. However, the disruption of genes with homology to *comEA* and *comEC* in L-forms of *K. viridifaciens* had no effect on their ability to take up DNA, suggesting a mechanism independent of the canonical natural genetic transformation machinery.

Endocytosis is a fundamental and highly regulated process in eukaryotes that is involved in the uptake of nutrients, regulation of plasma membrane composition, sensing of the extracellular environment and signaling^43^. Invagination of the membrane and subsequent membrane scission and vesicle formation allows cells to internalize a wide array of cargo such as fluids, ligands, plasma membrane proteins, and sometimes even entire bacteria. Invagination is often followed by passing the cargo through the endosomal pathway and lysosomal degradation^26^. Specific mammalian cells can take up DNA, followed by active gene expression^44^, which potentially occurs via endocytosis, although the exact mechanism is unclear^45^. This work shows that L-forms use an endocytosis-like mechanism for the uptake of DNA, whereby membrane invagination leads to the formation of intracellular vesicles that, during their formation, encapsulated extracellular material (Fig. 5). Via this process, not only DNA but also other macromolecules such as 3 kDa dextran and even 150-nm lipid nanoparticles were encapsulated, strongly suggesting that the uptake process is non-specific. The uptake process is inhibited by conditions that reduce metabolic activity and energy production. Interestingly, an older study also reports the uptake of fluorescent dextrans in internal vesicles of *Bacillus subtilis* L-forms, which was proposed to occur via fluid-phase endocytosis^46^.

The exact mechanism underlying the formation of intracellular vesicles in L-forms is unknown but may depend on increased membrane dynamics due to excess membrane synthesis^6,47^. An imbalance in the cell surface-to-volume ratio due to excess membrane synthesis can lead to internal vesicle formation in spherical *E. coli* and *B. subtilis* shape mutants^6,48^. Internal vesicles or vacuoles can also be formed in enlarged protoplasts and spheroplasts (the latter containing an outer membrane) which are maintained in conditions that allow cell membrane expansion^49,50^. Indeed, a lack of excess membrane production may also explain why we did not observe consistent DNA uptake in protoplasts and S-cells, both of which are unable to proliferate without their wall. However, we cannot exclude whether proteins similar to those involved in eukaryotic endocytosis, such as coat proteins, scission machinery, or cytoskeletal proteins^43^, are involved in the formation of internal vesicles in L-forms. However, it must be noted that *B. subtilis* L-forms do not rely on known cytoskeletal proteins for membrane deformation and proliferation^51^.

High-resolution electron microscopy imaging revealed multiple internal vesicles inside L-form cells. Interestingly, the L-forms also contained regions not surrounded by a membrane but were lined with darker spots. These dark spots, which are generated by the local charging of the electrons with the material, were previously described as proteinaceous or lipid bodies^38,39^, and may possibly originate from the degradation products of the membrane of internal vesicles. The likely disintegration of internal vesicles was also captured using timelapse imaging. This disintegration would lead to release of the vesicle cargo into the cytoplasm. In eukaryotes, escape of therapeutics from endosomal vesicles can be mediated by bacterial, viral, and chemical agents or by nanoparticles^52,53^. Escape mechanisms include pore formation, destabilization of the membrane, nanoparticle swelling, or osmotic rupture. High sucrose levels or the proton sponge effect facilitate the influx of protons followed by chloride ion accumulation and inflow of water, leading to rupture of the vesicle^54,55^. Acidification of endosomes occurs via membrane-localized vacuolar ATPases (V-ATPases) that pump protons into the vesicles^56^. Bacteria have similar proton pumps called F-ATPases on their plasma membrane and have been found on the membrane of intracellular vesicles of enlarged protoplasts^57^. Considering the complexity of known escape mechanisms, further research is required to understand how internal L-form vesicles can disintegrate to release their contents into the cytoplasm.

Modern life forms are complex biological systems, which likely evolved from much simpler cells. Two model systems to study putative early life forms are giant lipid vesicles and L-forms due to their lack of a cell wall and biophysical way of proliferation^8,9^. Horizontal gene transfer is thought to have played a pivotal role in the evolution of early life^58^. This may have occurred in cells that did not yet evolve a cell wall, allowing genetic recombination after cell fusion or lightning-triggered electroporation^59,60^, yet other mechanisms of HGT were unknown. Internal vesicles have been observed in L-forms of other bacterial species, with varying functions and mechanisms of vesicle formation described^61,62^. L-forms of *Listeria monocytogenes* are capable of forming DNA-containing internal vesicles along the inside of the cell membrane, which upon release become metabolically active^63,64^, as well as form internal vesicles via membrane invagination, which likely contain extracellular medium^47^. Additionally, secondary invagination of the vesicle membrane itself can result in vesicles containing cytoplasm and represent viable offspring.

Interestingly, preliminary work shows that L-forms of *L. monocytogenes* also have the ability to take up extracellular plasmid DNA and become transformed without the use of PEG^65^. This bacterial species is not known to be naturally transformable and does not contain a functional competence system. This suggests that a similar endocytosis-like mechanism as observed in L-forms of *K. viridifaciens* could be responsible for DNA uptake. These examples provide additional support for the existence of bacterial endocytosis.

A recent study reports similarities between spherical microfossils and wall-less protoplasts^66^. When protoplasts were grown in conditions that mimic the presumed conditions of the Archean Eon, the period when life formed on Earth, intracellular vesicles were formed. These vesicles were also observed in 3.5-2.4-billion-year-old microfossils, suggesting that wall-less cells may have been present in ancient times. However, it should be noted that wall-less cells can also be formed from walled bacteria. For example, wall-less *Mycoplasma* have been shown to be derived from walled bacteria via degenerative evolution^67^, and L-forms generated in the lab also originate from walled cells. L-forms may not have been the primordial cells themselves, but rather function as a model to study putative early life forms. Therefore, we propose that the endocytosis-like process observed in L-forms reflects an ancient mechanism of how primordial cells may have acquired new genetic material and nutrients via engulfment before the invention of the cell wall.

In conclusion, our work shows that the permanent loss of the bacterial cell wall allows the uptake of DNA, dextran, and 150-nm-sized lipid nanoparticles via internal vesicle formation. The invagination of the cell membrane leads to the engulfment of external fluids and subsequent vesicle formation. Eventually, the vesicle may disrupt, resulting in the release of the cargo into the cytoplasm. This is an energy-dependent process that has similarities to a simple form of endocytosis as seen in eukaryotes. Future studies are required to further understand the molecular mechanisms behind this process.

## Methods

### Bacterial strains and culture conditions

The bacterial strains and plasmids used in this study are listed in Supplementary Tables 4 and 5, respectively. Bacterial cell lines are available from the corresponding authors upon reasonable request. *Kitasatospora viridifaciens* DSM40239^68^ was grown confluently on maltose-yeast extract medium (MYM) to obtain spores, which were harvested after 3–4 days of growth^69^. In brief, spores were resuspended in MilliQ using a cotton swab and filtered through a syringe filled with cotton wool. Spores were resuspended in 20% (v/v) glycerol (G1345, Duchefa Biochemie) and stored at −80 °C until use. For mycelial growth in liquid, *K. viridifaciens* was grown overnight at a density of 1 × 10^6^ spores ml^−1^ in L-phase broth (LPB) without sucrose at 200 rpm. Strains were grown for 2 days in LPB with sucrose (S0809, Duchefa Biochemie) at 100 rpm to induce the formation of S-cells^7^. L-forms were grown on solid L-phase medium agar (LPMA) or liquid LPB^7^. Liquid cultures were inoculated with spores for *K. viridifaciens* strains or with a frozen aliquot of a 1- to 2-day-old L-form culture in case of L-form strains. L-forms were grown in liquid culture for 3–4 days for chemical transformation and 7 days for all other experiments unless stated specifically. L-forms were adjusted to 5–7.5 × 10^7^ CFU ml^−1^ for transformation assays (based on OD_600_ of 3 for 3- and 7-day-old cells and 0.2 for 1-day-old cells), and 2.5–5 × 10^7^ CFU ml^−1^ (OD_600_ of 2) for all other experiments with 7-day-old cells. All *Kitasatospora* cultures were grown at 30 °C.

Where necessary, antibiotics (100 µg ml^−1^ ampicillin, K029, Roth; 25 µg ml^−1^ chloramphenicol, C0113; Duchefa Biochemie; 5 µg ml^−1^ thiostrepton, 598226, Calbiochem; 50 µg ml^−1^ apramycin, A0164, Duchefa Biochemie; 100 µg ml^−1^ hygromycin B, K547, Amresco, with the exception of 200 µg ml^−1^ hygromycin B for LB medium) were added to the culture medium. *Escherichia coli* strains were grown on solid or liquid LB medium (while shaking at 250 rpm) at 37 °C. *E. coli* JM109^70^ was used for cloning purposes and to obtain methylated plasmid DNA, while *E. coli* ET12567/pUZ8002^71^ was used to obtain methylation-deficient DNA.

### Construction of plasmids

All PCRs were performed using PFU or Q5® High-Fidelity DNA polymerase (NEB). The primers used in this study are listed in Supplementary Table 6. GeneRuler DNA Ladder Mix (SM0334, Thermo Scientific) was used to confirm the size of DNA molecules via gel electrophoresis. To create pFL-*ssgB* (Supplementary Table 5), a hygromycin resistance cassette was amplified using primer pair Hyg_F-231_EEV and Hyg_R + 1237_HEV with pMS82^72^ as the template. The PCR products were digested with EcoRV and cloned into pWHM3-oriT^73^ to generate pWHM3-oriT-hyg (Supplementary Table 5). The 3’ flank of *ssgB* was digested from pKR1^7^ and cloned into pWHM3-oriT-hyg using XbaI and HindIII to generate the final plasmid. All restriction enzymes were ordered from New England Biolabs.

pRK1 (Supplementary Table 5) was created by amplifying the upstream flanking region of *comEA* by PCR with primers FL1-comEA/comEC-FW and FL1-comEA/comEC-REV, thereby introducing unique EcoRI and XbaI restriction sites, while the downstream flanking region of *comEC*, made by gene synthesis (Baseclear, Leiden, the Netherlands) was flanked by XbaI and HindIII sites. The flanking regions and apramycin cassette were cloned in pWHM3-oriT using the EcoRI, HindIII restriction sites interspersed with an apramycin resistance cassette containing flanking XbaI sites, thereby creating the final plasmid. The *comEA/comEC* deletion mutant was created in L-form strain *alpha*^7^ using pRK1, which replaced the nucleotides +58 relative to the start codon of *comEA* (BOQ63_029625) until + 2489 relative to the start codon of *comEC* (BOQ63_029630) with an apramycin resistance cassette. Note that the gene annotation of *Streptomyces viridifaciens* ATTC11989 (accession CP023698) was used to determine the putative correct start and stop codons for *comEC*, resulting in the genome location 5,041,836 to 5,044,433 on CP090841.

To create pIJ82-GFP, the region containing the *eGFP* gene with a *gap1* promoter was amplified from pGreen^74^ using primer pair gap1_FW_BglII and egfp_RV_EcoRI. The resulting PCR product was cloned into pIJ82 using BglII and EcoRI to generate the final plasmid.

### Construction of bacterial strains

To create new L-form strains, transformation of *alpha* with plasmid DNA was achieved using chemical transformation based on polyethylene glycol (PEG)^10^ as described below. Plasmid DNA was isolated from *Escherichia coli* ET12567/pUZ8002 to obtain methylation-deficient DNA. L-form strains *alpha* pIJ82-GFP and *alpha*Δ*divIVA* pIJ82-GFP were created using chemical transformation of *alpha* and *alpha*Δ*divIVA* with pIJ82-GFP, respectively, followed by selection with hygromycin B (Supplementary Table 4). The strains were verified using the detection of fluorescent eGFP production using fluorescence microscopy. Strain *alpha*Δ*comEA/EC* was obtained by chemical transformation of *alpha* with pRK1 followed by selection for apramycin (Supplementary Table 4). Subsequent growth on a nonselective medium allowed for double homologous recombination leading to the replacement of the *comEA/EC* region by an apramycin resistance cassette, leading to thiostrepton-sensitive, apramycin-resistant cells. The strain was verified by PCR using primer pair ComEA_Apra_check_FW and ComEC_Apra_check_RV to confirm the replacement of the region by the apramycin cassette. To further confirm the deletion of this region, PCR was performed using primer pairs ComEC_Presence_Check_1_FW/RV and ComEC_Presence_Check_2_FW/RV, which amplify parts of *comEC* only if this genomic region is still present.

### Genomic DNA preparation

Genomic DNA was isolated from a 5-day-old culture of *alpha*Δ*ssgB*^7^ using phenol:chloroform extraction^10^. Briefly, the cell pellet was resuspended in 10.3% (w/v) sucrose containing 0.01 M ethylenediamine tetraacetic acid (EDTA, 20296.291, VWR Chemicals BDH) pH = 8, following lysis with 10% (w/v) sodium dodecyl sulfate (SDS, 20765.02, Serva). Extraction with phenol:chloroform (1:1 mix of phenol, 10001173, Fisher BioReagents^tm^ and chloroform, 32211, Honeywell) was performed and the nucleic acids were precipitated using isopropanol (33539, Honeywell). The pellet was dissolved in Tris-EDTA buffer (Trizma® base, RDD008, Sigma-Aldrich), followed by RNase A (EN0531, Thermo Fisher) and Proteinase K treatment (19131, Qiagen). The gDNA was isolated using phenol:chloroform extraction and precipitated using absolute ethanol (5250501, Biosolve) before resuspension in nuclease-free water. Fragmented gDNA was obtained by beat-beating the intact gDNA for 12 min using 2 mm diameter glass beads in a Mikro-Dismembrator U (Sartorius) at 2000 rpm. Chromosomal DNA concentrations were verified using the Quant-IT™ Broad-Range dsDNA Assay Kit (Q33130, Invitrogen).

### Preparation of protoplasts from *Kitasatospora*

*K. viridifaciens* strain DSM40239 was inoculated at a density of 5 × 10^6^ spores ml^−1^ in TSBS:YEME (1:1) liquid medium with 0.5% (w/v) glycine (G0709, Duchefa Biochemie) and 5 mM MgCl_2_ (M0533, Duchefa Biochemie). The culture was grown for 48 h while shaking at 200 rpm, after which protoplasts were prepared^10^. Cultures of 72 h were used for *K. viridifaciens* pIJ82-GFP and *K. viridifaciens* pRed*. Cells were washed with 10.3% (w/v) sucrose before lysozyme treatment was performed by the addition of 10 mg ml^−1^ of chicken egg-white lysozyme (~ 70,000 U mg^−1^, 62971, Sigma-Aldrich). The cells were incubated for 2–3 h at 100 rpm and 30 °C, after which mycelial fragments were separated from the protoplasts by filtration through a cotton wool filter. Cells were concentrated by centrifugation at 1000×*g* if required.

### Isolation of S-cells from *Kitasatospora*

S-cells were isolated from LPB cultures by filtration^7^. In short, the culture was filtered through a sterile EcoCloth™ filter (AMEC0003, Contec) and subsequently passed through a 5 µm Isopore™ membrane filter (TMTP01300, Merck). The cells were concentrated by gentle centrifugation at 1000×*g* for 20 min, after which 90% of the supernatant was removed. The cell pellet was resuspended carefully in the remaining liquid. For testing a high concentration of S-cells for spontaneous DNA uptake, *K. viridifaciens* was inoculated at 1 × 10^7^ spores ml^−1^, and filtration was only performed through the EcoCloth™ filter.

### Chemical transformation

Freshly prepared protoplasts, S-cells, L-forms, or mycelial cells were kept on ice prior to transformation. For chemical transformation, 50 µl of cells were mixed with 1 µg pRed*^75^, 150 ng gDNA of strain *alpha*Δ*ssgB*, filter-sterilized salt-lysed cells (35 ng DNA from *alpha*Δ*ssgB*), or MilliQ. Then, 200 µl of 25% (w/v) PEG 1000 (14805-B, NBS Biologicals) in P-buffer^10^ was added to the cells, followed by gently mixing and diluting the suspension in P-buffer. Serial dilutions were plated on LPMA medium and after 16- to 18-h incubation, an overlay was performed with 1 ml of P-buffer containing antibiotics. Colony forming units (CFU) were counted after 7 days for L-forms and mycelium or after 14 days for S-cells and protoplasts. Transformants were verified by streaking on a selective medium and microscopy.

### Transformation assay

Freshly prepared cells were incubated with 30 ng µl^−1^ unmethylated DNA (pRed* or pFL-*ssgB* as indicated) or MilliQ for 18–24 h at 100 rpm unless stated otherwise. A final concentration of 100 or 10 ng µl^−1^ intact gDNA and 10 ng ul^−1^ for fragmented gDNA isolated from *alpha*Δ*ssgB* was used in combination with both 1- and 7-day-old *alpha*. Dilutions were plated on selective and nonselective LPMA after careful resuspension. Mycelial cells were diluted similarly on MYM medium. Colony forming units were determined after 7-day incubation at 30 °C for L-forms and mycelium and up to 14 days for protoplasts and S-cells. Transformants were verified by growth on a selective medium and by PCR (using primers Tsr_Hyg_FW1 and Tsr_Hyg_RV1) or microscopy. Cells were prepared from at least five replica cultures to compare transformation efficiencies between strains. DNA uptake of S-cells was tested using filtrate obtained via the standard procedure, as well as more concentrated filtrate that was obtained via inoculation of 1 × 10^7^ spores ml^−1^ and filtration of the bacterial culture through the EcoCloth™ filter only. Colony plates were imaged using the Epson Perfection V600 Photo scanner with Epson Scan Utility v3.9.2.0 software.

### Membrane fluidity

Three replicate cultures of 1-, 3-, and 7-day-old L-forms or freshly prepared protoplasts were subjected to a Laurdan dye assay as a measure for membrane fluidity^23^. About 1 ml of each culture was first centrifuged at 1000×*g* for 10 min to remove any traces of the culture media. Cells were resuspended in 1-ml P-buffer and adjusted to an OD_600_ of 0.6. 10 mM Laurdan (6-Dodecanoyl-2-Dimethylaminonapthalene, D250, Invitrogen) stock solution was prepared in 100% dimethylformamide (DMF, D4551, Sigma-Aldrich) and stored at −20 °C in an amber tube. To each 1 ml OD-adjusted culture, 1 µl of Laurdan dye was added to a final concentration of 10 µM. The cultures were then incubated in the dark at 30 °C for 10 min, while shaking at 100 rpm. The cells were washed three times with P-buffer containing 1% dimethyl sulfoxide (DMSO, 41639, Sigma-Aldrich) to remove unbound dye molecules before the cells were resuspended in P-buffer. About 200 µl of this resuspended culture was aliquoted into a 96-well black/glass bottom SensoPlate™ (655892, Greiner Bio-One). Three technical replicas were measured per culture, as well as one replica per culture condition without dye to measure background fluorescence.

Sample excitation was performed at 350 nm followed by fluorescence emission capture at 435 and 490 nm, determined using a Spark® multimode microplate reader (Tecan) with Sparkcontrol V3.1 software. After subtracting the background fluorescence, the generalized polarization (GP) value was calculated using Eq. (1):

1 $${{{{{\rm{GP}}}}}}=\frac{{I}_{435}-{I}_{490}}{{I}_{435}+{I}_{490}}$$

Values obtained after calculation lie in the range of −1 to +1 with those closer to −1 indicating greater fluidity.

Preparation of cells for quantification of membrane fluidity by microscopy was performed as following. Cells were washed and OD-adjusted as mentioned above. Laurdan dye (stock concentration 10 mM) was added to 100 µl of culture to get a final concentration of 100 µM. The culture was placed at 30 °C for 5 min, while shaking at 100 rpm in the dark. About 900 µl of prewarmed P-buffer containing 1% DMSO was added and the culture was centrifuged (1000×*g*, 10 min) to remove any unbound dye molecules. The cells were finally resuspended in 100 µl of P-buffer for microscopy analysis. Cells treated similarly but without Laurdan dye were used as control for microscopy measurements.

### Preparation of fluorescently labeled DNA

Fluorescently labeled plasmid DNA was prepared using The Mirus Label IT® Cy™5 Labeling Kit (MIR 2725) according to the manufacturer’s specifications. Aliquots of labeled DNA (100 ng µl^−1^) were stored at −20 °C until further use.

### Self-assembly of lipid nanoparticles

All lipids (DLin-MC3-DMA^76^; Cholesterol, C8667, Sigma-Aldrich; Avanti Polar Lipids: DSPC, 850365, DMG-PEG2000, 880151, 18:1 Liss Rhod PE, 810150) were combined in a molar ratio of 50/38.3/10/1.5/0.2 using stock solutions (100 µM–10 mM) in chloroform:methanol (1:1 mix of chloroform, 22706, VWR Chemicals, and methanol, 83638, VWR Chemicals). Organic solvents were evaporated under a nitrogen stream and the remaining solvent was removed in vacuo for at least 1 h. Subsequently, the lipid film was dissolved in EtOH_abs_ (20821, VWR Chemicals) and a 50 mM citrate buffer (pH = 4, MilliQ; using citric acid, C0759, and sodium citrate tribasic dehydrate, C7254; Sigma-Aldrich) was prepared. Each solution was loaded into separate syringes and connected to a T-junction microfluidic mixer. The solutions were mixed in a 3:1 flow ratio of citrate buffer against lipids (1.5 mL min^−1^ for citrate buffer, 0.5 mL min^−1^ for lipid solutions), giving a total lipid concentration of 1 mM. After mixing, the solution was directly loaded in a 10k MWCO dialysis cassette (Slide-A-Lyzer™, Thermo Scientific) and dialyzed overnight against 1× Phosphate Buffered Saline (PBS), containing 137 mM NaCl (NAC02, Formedium), 2.7 mM KCl (1.04936, VWR Chemicals), 8 mM Na_2_HPO_4_ (1.06586, VWR Chemicals), and 2 mM KH_2_PO_4_ (60229, Sigma-Aldrich), overnight. Lipid nanoparticles (LNP-LR) are available from the corresponding authors on reasonable request. All incubations with LNPs were performed with cells resuspended in LPB medium, of which the final volume of LNP solution was 25%.

### Hydrodynamic diameter and zeta-potential measurements

Preparations of lipid nanoparticles were characterized in the following manner (Supplementary Table 8). Dynamic light scattering (DLS) measurements were performed on a Zetasizer Nano Series S (Malvern Instruments, Malvern, UK). The incorporated HeNe laser works at a wavelength of 633 nm and uses a detector at an angle of 173° (noninvasive backscatter technology). Measurements were recorded with 1 min equilibration time in UV cuvettes at 25 °C. For the estimation of z-average diameter (intensity weight mean diameter) and polydispersity index (PDI) (relative width of particle size distribution), samples were prepared by tenfold dilution with 1× PBS. For the estimation of the zeta potential, the sample was diluted with 0.1× PBS and measured in a Zetasizer Nano Series SZ (Malvern Instruments, Malvern, UK). All the data were in triplicates to obtain the mean value.

### Fluorescence and light microscopy

Detection of fluorescence emission of transformants was performed using a Zeiss Axioscope A.1 equipped with a Zeiss Axiocam 305 color digital camera, using filter set 63 HE (Carl Zeiss, consisting of a 572/25 nm bandpass excitation filter, 590 nm beamsplitter and 629/62 nm bandpass emission filter) to capture mCherry fluorescence. Single colonies were imaged using a Zeiss SteREO Discovery v. 8 equipped with a Schott VisiLED Ring Light S80-55 and Bresser MikroCam SP5.0. Bresser MikroCamLabII software was used to capture images. All other microscopy was performed using a Zeiss LSM 900 confocal microscope with Airyscan 2 module, temperature control chamber, and Zeiss Zen 3.1 software (blue edition, Carl Zeiss Microscopy GmbH) unless specified otherwise. All excitation and emission settings for this microscope are listed in Supplementary Table 7. Multichannel (DIC and fluorescence) and multistack images were obtained unless specified otherwise. 10 μl of cells were imaged on an 8-chamber slide (ibidi®) coated with 0.1% (w/v) poly-L-lysine (P8920, Sigma-Aldrich; excess poly-l-lysine was removed and the slide was allowed to dry prior to applying the sample). For timelapse imaging or overnight incubation in the temperature control chamber, 400 μl of cell culture added to a 35 mm imaging μ-Dish (ibidi®) and allowed to settle at 30 °C for one hour before overnight imaging. Image analysis was performed using Fiji (ImageJ) software^77^.

Chromosomal DNA was visualized after incubation for 30 min with SYTO 9 (S34854, Invitrogen) at a final concentration of 2 μM. Cell membranes were visualized by incubation with SynapseRed C2M (SynapseRed, PK-CA707-70028, PromoKine, PromoCell GmbH) at a final concentration of 40 μg ml^−1^. After overnight incubation in a μ-Dish (ibidi®) using the Zeiss LSM 900 confocal temperature control chamber, cells were imaged using the Airyscan mode with super-resolution post-image processing via the Zen software. Protoplasts and S-cells were incubated with SynapseRed up to 72 h before imaging on a glass slide. Quantification of putative internal vesicles was performed after incubating 7-day-old L-forms (*alpha* pRed*) or freshly harvested S-cells and protoplasts (*K. viridifaciens* pRed*) producing cytoplasmic mCherry with SynapseRed for 0 or 72 h. SYTO 9 was added directly before imaging to identify DNA. Cells were placed on an eight-chamber slide (ibidi®) coated with 0.1% poly-l-lysine. L-forms and S-cells were imaged from top to bottom with a step size of 0.5 μm and protoplasts with a step size of 0.28 μm to account for their smaller cell size. Cells having one or more regions lacking mCherry, SYTO 9 or SynapseRed staining were counted as a cell with a putative internal vesicle using the Cell Counter plugin in Fiji (ImageJ).

Uptake of fluorescently labeled DNA was assessed by incubating cells with Cy5-labeled plasmid DNA (pFL-*ssgB*) at a final concentration of 1.25 μg ml^−1^ and was imaged in a μ-Dish (ibidi®) after 72 h.

To capture internal vesicle formation and uptake of Dextran-Texas Red (D-TR, D-3329, 3000 MW, neutral, Molecular Probes), cells of *alpha* pKR2 were incubated with a final concentration of 1 mg ml^−1^ D-TR in PBS and were imaged overnight. Multistack imaging across 6 μm total distance with 1.5 μm steps was done with an image captured every 10 min. Imaging of D-TR uptake in L-forms, protoplasts, or S-cells was performed after incubation up to 72 h. Quantification of the percentage of cells that had taken up D-TR was performed as follows. Cells producing cytoplasmic eGFP were incubated with PBS or 1 mg ml^−1^ D-TR in duplo for 72 h (7-day-old *alpha* pIJ82-GFP or freshly harvested S-cells or protoplasts from *K. viridifaciens* pIJ82-GFP). Cells were diluted ten times in LPB medium and gently centrifuged for 10 min at 1000×*g*, after which the supernatant was replaced by LPB medium. Cells were placed on an eight-chamber slide (ibidi®) coated with 0.1% poly-l-lysine. Z-stack images were acquired from top to bottom of the cells with 0.28 μm steps. Cells with putative D-TR uptake were identified as those lacking a region of cytoplasmic eGFP (putative internal vesicle) while revealing an increased D-TR emission at this region as measured using the Plot Profile tool in Fiji (ImageJ). Cells with and without uptake were counted using the Cell Counter plugin in Fiji (ImageJ).

Uptake of red fluorescent LNPs (LNP-LR) by *alpha* was visualized by imaging after overnight incubation in a μ-Dish (ibidi®) or after incubation for up to 3 days prior to imaging as indicated. Inhibition of LNP uptake was performed by incubation in the presence of 1, 2.5, or 10 mM sodium azide (S-8032, Sigma-Aldrich) or incubation at 4 °C, and images were obtained via the Zen software after 0, 24, and 48 h. To determine the subcellular localization of LNP-LR in *alpha* pIJ82-GFP, imaging was performed using the Airyscan mode with super-resolution post-image processing and analyzed using the pixel intensity of the red (LNP-LR) and green (eGFP) channels using the Plot Profile tool in Fiji (ImageJ).

To measure the membrane fluidity, samples were excited using a 405 nm laser and images were captured at emissions of 430 and 500 nm. GP value was calculated using the Calculate GP plugin in Fiji^78^ to obtain a histogram of pixel counts over the range of −1 to +1. Briefly, the image is split into individual channels, followed by background subtraction and setting of the non-significant pixels to zero. The images are then assigned letters A and B to calculate A − B and A + B using the image calculator. Finally, a ratio of (A − B)/(A + B) is shown as an image where minimum pixel values are set to −1 (red) and maximum pixel values set to +1 (blue). Using the analyze histogram function, a list of values is obtained and used for plotting the distributions of different samples.

To capture vesicle disruption, L-forms were resuspended in fresh LPB to an OD_600_ of 0.04. Dilutions were placed in a 96-wells black/glass bottom SensoPlate and gently spun down for 5 min at 1000×*g* to settle the cells at the bottom of the wells. Cells were imaged using a Lionheart FX automated microscope (BioTek) with Gen 5 v.3.10 software at a magnification of 60× air (brightfield and mCherry using Texas Red 586/647 filter cube). Z-stack images were captured every 15 min with a step size of 1 µm covering 12 µm total for 20 h (Supplementary Movie 5) or a step size of 0.5 µm covering 5 µm total for 17.5 h (Supplementary Movies 6, 7).

### Cryo-correlative fluorescence and electron microscopy

#### High-pressure freezing

Seven-day-old L-form strain *alpha* pIJ82-GFP expressing cytoplasmic eGFP was adjusted to OD_600_ of 2 in fresh medium containing 25% (v/v) PBS and a final concentration of 17% (w/v) sucrose. Cells were incubated for 4 days, during which cells settled to the bottom. A few microliters of the resuspended L-form pellet was sandwiched between HPF (high-pressure-freezing) carriers with 2 mm internal diameter (either 0.1 mm or 0.05 mm cavity, Art. 241 and Art. 390 respectively, Wohlwend) and tailor-made grid labeled, flat-sided finderTOP (Alu-platelet labeled, 0.3 mm, Art.1644 Wohlwend) to allow an imprint of a finder matrix on the amorphous ice^79^. The finderTOP was treated with 1% l-α-phosphatidylcholine (61755, Sigma-Aldrich) in ethanol (1.00983.1000, Supelco) before freezing. The samples were then high-pressure frozen (Live µ, CryoCapCell) and stored in liquid nitrogen until imaging.

To improve the correlation between cryo-light and cryo-electron microscopy, the frozen samples were loaded into a universal cryo-holder (Art. 349559-8100-020, Zeiss cryo accessory kit) using the ZEISS Correlative Cryo Workflow solution, fit into the PrepDek® (PP3010Z, Quorum technologies, Laughton, UK). Here, the HPF carriers fits into a universal cryo-holder, which subsequently can be placed into an adapter specific for cryo-light or cryo-electron microscopy.

#### Cryo-fluorescence imaging to detect regions of interest (ROI)

The frozen samples were imaged with a cryo-stage adapter (CMS-196, Linkam scientific inc.) applied to an upright confocal microscope (LSM 900, Zeiss microscopy GmbH) equipped with an Airyscan 2 detector. Overview images (Zeiss C Epiplan-Apochromat 5×/0.2 DIC) were made with reflection microscopy to visualize the gridded pattern on the ice surface. Next, medium-resolution Z-stack images (Zeiss C Epiplan-Apochromat 10×/0.4 DIC) were taken with a 488 nm laser (0.4%) with a voxel size of 0.15 µm × 0.15 µm × 1.18 µm. Using this resolution, cells of interest could be selected and Z-stack images were created (Zeiss C Epiplan-Neofluar 100x/0.75 DIC) using a 488 nm laser (4%), with a voxel size of 0.08 µm × 0.08 µm × 0.44 µm. In addition, the ice surface was imaged in all ROIs with reflection microscopy for correlation purposes in the FIB-SEM.

Prior to cryo-light imaging, a Zeiss ZEN Connect project (Zeiss software for correlative microscopy, version 3.1) was created to make a working sheet (canvas) to align and overlay all the images and to facilitate further correlation with cryo-FIB-SEM.

#### 3D Cryo-FIB-SEM

The sample was sputter-coated with platinum, 5 mA current for 30 s, using the prep stage sputter coater (PP3010, Quorum technologies, Laughton, England) and was transferred into the Zeiss Crossbeam 550 FIB-SEM (Carl Zeiss Microscopy GmbH, Oberkochen, Germany) using the PP3010T preparation chamber (Quorum, Laughton, England). Throughout imaging, the samples were kept at −140 °C and the system vacuum pressure was 1 × 10^−6^ mbar.

After inserting the sample into the FIB-SEM chamber, overview images were taken using the SEM to align the data with the LSM reflection image of the surface of the same ZEN Connect project. This alignment enables the stage registration, which allows using the fluorescence signal to navigate to different regions of interest. After initial alignment using the SEM, a FIB image of the surface was collected with the 30 kV@10 pA probe at 54° tilt.

A coarse trench was milled for SEM observation using the 30 kV@30 nA FIB probe. The cold deposition was done with platinum for 30 s. Fine FIB milling on the cross-section was done using the 30 kV@700 pA probe. For serial FIB milling and SEM imaging the slice (trench) width was 40 μm and for FIB milling the 30 kV@300 pA probe was used, with a slice thickness of 20 nm. When a new slice surface was exposed by FIB milling, an InLens secondary and EsB images were simultaneously collected at 2.33 kV acceleration potential with 250 pA probe current. The EsB grid was set to −928 V. The image size was set to 2048 × 1536 pixels. For noise reduction line average with a line average count *N* = 46 at scan speed 1 was used. The voxel size of all stacks was 5 nm^3^ × 5 nm^3^ × 20 nm^3^.

#### 3D FIB-SEM image post-processing

The cryo-FIB-SEM images were processed using MATLAB (R2018b, Natick, Massachusetts: The MathWorks Inc.) to correct for defects such as curtaining, misalignment, and local charging. The same software was used for subsequent noise reduction and contrast enhancement. A summary of each processing step is as following:

##### Curtaining

Removing the vertical stripes in the stacks was done following a wavelet-FFT filtering approach^80^. In brief, the high-frequency information corresponding to the vertical stripes was successively condensed into a single coefficient map using decomposition by the coif wavelet family. Subsequently, a 2D-fourier transform was performed to further tighten the stripe information into narrow bands. Finally, the condensed stipe information was eliminated by multiplication with a gaussian damping function and the destriped image was reconstructed by inverse wavelet transform.

##### Alignment

The consecutive slices were aligned using normalized cross-correlation. Briefly, the first image in the stack was chosen as the reference and the second image was translated pixel by pixel across the reference and a normalized cross-correlation matrix was obtained using the normxcorr2 function. The location of the highest peak in the cross-correlation matrix (representing the best correlation) was then used to calculate the translation required to align the two images. Once the moving image was aligned with the reference image, it served as the reference for the alignment of the subsequent slice.

##### Charging

Elimination of the local charge imbalance was achieved using anisotropic gaussian background subtraction. Briefly, the imgaussfilt function was used to perform 2D-gaussian smoothing with a two-element standard deviation vector. The elements in the vector were chosen in a manner to apply a broad and sharp gaussian in the horizontal and vertical directions, respectively. Subsequently, the corrected image was obtained by subtracting the filtered image from the original image.

##### Noise reduction

In order to improve the signal-to-noise ratio, noise reduction was performed using anisotropic diffusion filtering^81^. Briefly, using the imdiffuseest function, the optimal gradient threshold and the number of iterations required to filter each image was estimated. Subsequently, the imdiffusefilt function was applied with the estimated optimal parameter values to denoise each image.

##### Contrast enhancement

As the final processing step, the contrast was enhanced using Contrast-limited adaptive histogram equalization^82^. Using the adapthisteq function, the contrast was enhanced in two steps, using a uniform distribution and a low clipping limit in order to avoid over-amplification of homogeneous regions.

##### 3D segmentation

DragonflyTM image analysis and deep-learning software (version 2021.1, Objects Research Systems, Montreal, QC, Canada) was used to segment all image data.

### Bioinformatic search for putative competence genes

Protein sequences from *Bacillus subtilis* str. 168, *Neisseria gonorrhoeae*, and *Helicobacter pylori* strain P12 were obtained from the UniProt database or literature and are provided in Supplementary Data 1. Protein BLAST was run for these sequences against the translated coding sequence database of *Streptomyces viridifaciens* strain DSM40239 (also known as *K. viridifaciens* strain DSM40239), with sequence accession numbers CP090840, CP090841, and CP090842, using the offline BLAST software (v. 2.12.0). Hits with an E-value of 1 × 10^−6^ or lower were collected (Supplementary Table 1).

### Statistics

All statistics are stated and were performed using SPSS statistics software (IBM, version 27.0) except the two-proportion *z*-test, which was calculated manually. *P* values less than 0.05 were considered statistically significant. Test assumptions were determined using the following methods. Homogeneity of variances was tested using Levene’s test. Normality was tested using the Kolmogorov–Smirnov test, Shapiro–Wilk test, and Q-Q plots where applicable. Graphs were generated using Graphpad Prism v. 9.0.0 or using R version 3.6.1, and other graphical images were generated using Adobe Illustrator v. 26.3.1 or via Biorender.com (accessed in August 2022). Standard deviations were calculated and plotted via Graphpad Prism.

### Reporting summary

Further information on research design is available in the Nature Research Reporting Summary linked to this article.

## Supplementary information

Supplementary Information

Description of Additional Supplementary Files

Supplementary Data 1

Supplementary Movie 1

Supplementary Movie 2

Supplementary Movie 3

Supplementary Movie 4

Supplementary Movie 5

Supplementary Movie 6

Supplementary Movie 7

Reporting Summary

## Data Availability

Protein sequences obtained from the UniProt database or literature to perform the NCBI BLAST search are provided with accession numbers in Supplementary Data 1. All fluorescence and FIB-SEM micrographs in this paper, including the raw FIB-SEM data underlying the 3D segmentation volume rendering (Supplementary Movies 3, 4), as well as the micrographs used for vesicle and D-TR uptake quantification in Fig. 3f and Supplementary Tables 2 and 3, have been deposited in the Open Science Framework (OSF) database available at 10.17605/OSF.IO/5WKGJ. For the BlastP search, hits were collected from *Streptomyces viridifaciens* strain DSM40239 with accession numbers CP090840, CP090841, and CP090842. *Streptomyces viridifaciens* ATTC11989 (accession CP023698 was used to deduce the putative *K. viridifaciens comEC* start and stop codon for the gene knockout construct. Source data are provided with this paper.

## Source data

Source Data
