# Supplementary material for: Endocytosis-like DNA uptake by cell wall-deficient bacteria
Source: Nat Commun. 2022 Sep 22;13:5524. doi: 10.1038/s41467-022-33054-w (PMC9500057; doi:10.1038/s41467-022-33054-w)
Supplement: Supplementary file 1 — Supplementary Information [file 41467_2022_33054_MOESM1_ESM.pdf]

Supplementary Information

Endocytosis-like DNA uptake by cell wall-deficient bacteria

Renée Kapteijn[1], Shraddha Shitut[1], Dennis Aschmann[2], Le Zhang[1], Marit de Beer[3], Deniz Daviran[3], Rona Roverts[3], Anat Akiva[3], Gilles P. van Wezel[1], Alexander Kros[2], Dennis Claessen[1]

[1]Institute of Biology, Leiden University, Sylviusweg 72, 2333 BE Leiden, The Netherlands.

[2]Department of Supramolecular and Biomaterials Chemistry, Leiden Institute of Chemistry, Leiden University, Einsteinweg 55, 2333 CC Leiden, The Netherlands

[3]Electron Microscopy Center, Radboudumc Technology Center Microscopy, Nijmegen, The Netherlands

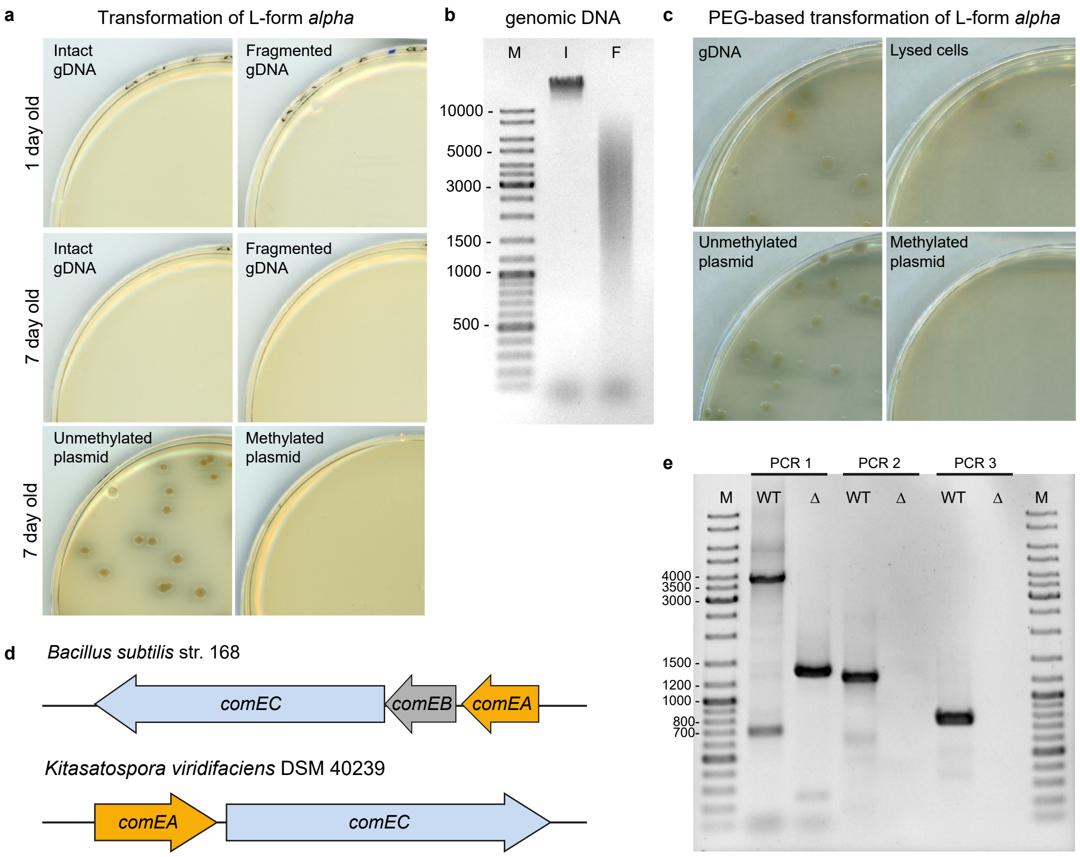

Supplementary Fig. 1. Analysis of transformation of cell-wall deficient cells and *alphaΔcomEA/EC* mutant.

a Transformation plates showing absence of transformation after 24-h incubation of 1-and 7-day-old L-form *alpha* with intact or fragmented (10 ng µl^{-1}) genomic DNA (gDNA) of *alphaΔssgB* containing an apramycin resistance cassette (*n* = *1* for each combination). In addition, whereas transformants are obtained using unmethylated plasmid DNA (pRed*, *n* = 1), this is not achieved using methylated DNA (*n* = *3*).
b Gel electrograph of 100 ng intact (I) or fragmented (F) gDNA of *alphaΔssgB* as used in the transformation assay in (a). One batch of gDNA was prepared, analysed and used for transformation. M = GeneRuler DNA Ladder Mix (Thermo Scientific) with DNA size given in bp.
c PEG-based transformation of *alpha* using unmethylated or methylated plasmid DNA (pRed*), gDNA or filter-sterilized salt-lysed cells from mutant line *alphaΔssgB* (*n* = *1*). Note that use of methylated DNA inhibited transformation as compared to unmethylated DNA.
d Localization of putative ComEA and ComEC-encoding genes (BOQ63_029625 and BOQ63_029630, respectively) on the chromosome of *K. viridifaciens* DSM 40239 as compared to *comEA* and *comEC* of naturally transformable *Bacillus subtilis* str. 168.
e Gel electrograph of PCR products from three different PCR mixes to confirm the replacement of *comEA* and *comEC* by an apramycin resistance cassette. M = GeneRuler DNA Ladder Mix (Thermo Scientific) with DNA size given in bp; PCR template used is indicated by WT = gDNA *alpha* and Δ = gDNA *alphaΔcomEA/EC*. Expected products: PCR 1 WT = 3676 bp, mutant = 1294 bp; PCR 2 WT = 1197 bp, mutant = no amplification, PCR 3 WT = 745 bp, mutant = no amplification. Each PCR was performed once. Source data are provided as a Source Data file.

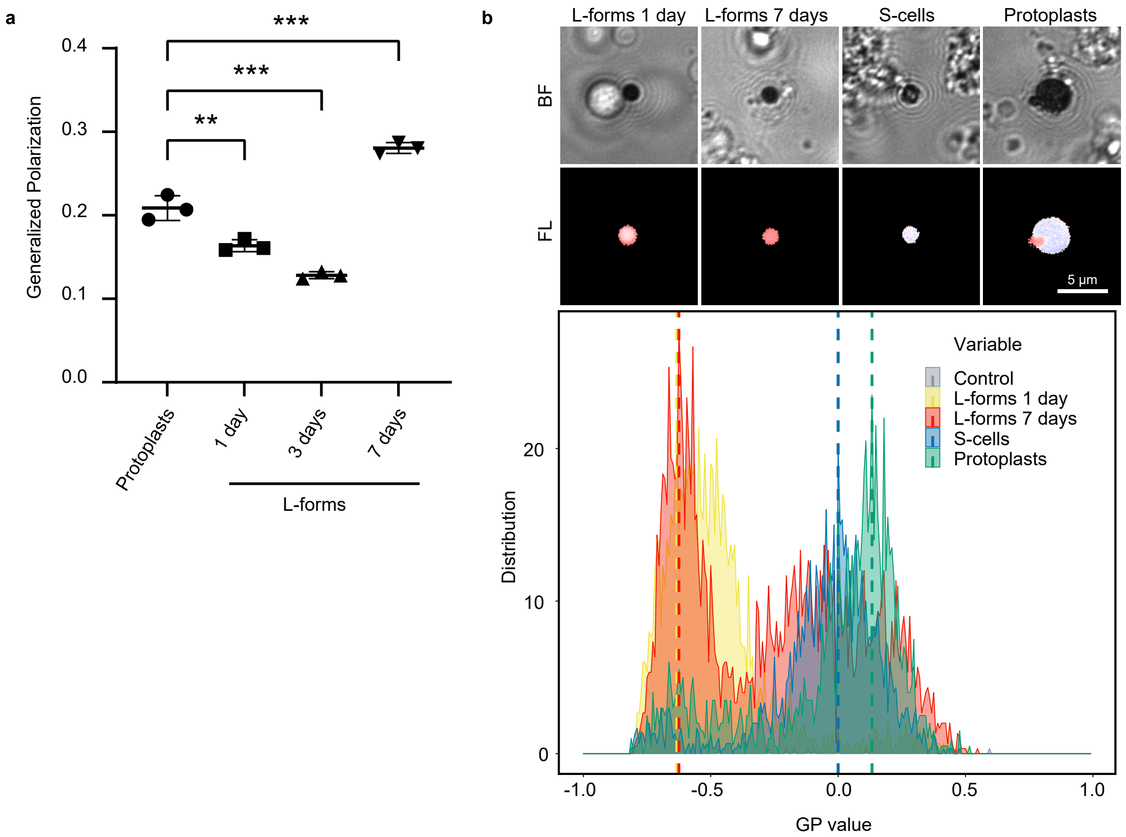

Supplementary Fig. 2. Membrane fluidity of cell-wall deficient cells.

a Generalized Polarization (GP) as measure of membrane fluidity of *K. viridifaciens* protoplasts and 1-, 3- and 7- day-old L-form cells (*alpha*). Lower GP indicates higher fluidity. All cell types were significantly different from each other, but this graph only highlights the difference between protoplasts and L-forms. Asterisks (** and ***) indicate $P \leq 0.01$ and $P \leq 0.001$, respectively (one-way ANOVA, F (3,8) = 154.81, P = 2.01e-07, Tukey post-hoc test: protoplasts and L-forms is P = 0.0014 (1 day); P = 2.30e-05 (3 days); P = 5.20e-05 (7 days) and L-form comparison between 1-3 day (P = 0.0064); 1-7 day (P = 1.00e-06); 3-7 day (P = 1.65e-07). Data are represented as mean ±SD with individual data points, n = 3 biological replicates.

b Membrane fluidity of L-form *alpha* (1- and 7-day-old), S-cells and protoplasts of *K. viridifaciens*. Cells from at least two cultures were combined during cell preparation, and three cell samples were imaged per cell type except for protoplasts (two samples) in one experiment. Top rows show brightfield images (BF) and heatmap of fluorescence emission (FL) (red to blue colour indicate GP values of -1.0 to 1.0 respectively) of representative cells stained with a Laurdan dye for quantifying the membrane fluidity. Bottom panel shows frequency distributions of the Generalized Polarization (GP) with the dashed line indicating the mode GP value. Lower GP values correspond to higher membrane fluidity indicating that L-forms have more fluid membranes compared to S-cells and protoplasts. Control = cells imaged and analysed without Laurdan staining. Source data are provided as a Source Data file.

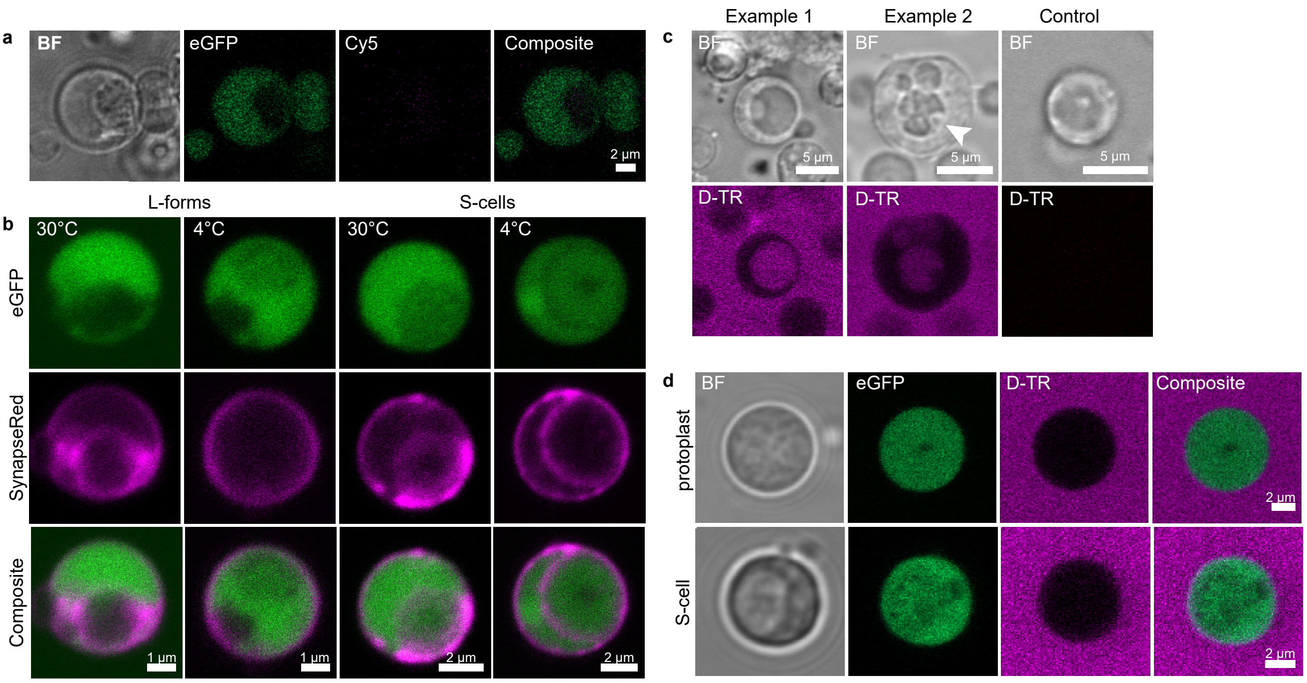

Supplementary Fig. 3. Comparison of SynapseRed staining and dextran uptake in wall-deficient cells, as well as imaging control for Cy5-DNA.

a Representative micrograph of *alpha* pIJ82-GFP incubated for 3 days without Cy5 DNA (Cy5, magenta) as fluorescence control (25 cells imaged from one incubation). BF = brightfield. **b** Representative images of 7-day-old L-form *alpha* pIJ82-GFP and S-cells of *K. viridifaciens* pIJ82-GFP after 3-day incubation with SynapseRed at 30 °C or 4 °C (a minimum of 5 observations per condition from two independent experiments). SynapseRed stains internal membranes at both temperatures in S-cells but only at 30 °C in L-forms. **c** *alpha* incubated with (example 1 and 2) or without (control) Dextran-Texas Red (D-TR; magenta) for 64 h, showing the formation of internal vesicles filled with D-TR (8 observations from one incubation). The arrow indicates the presence of a non-fluorescent secondary internal vesicle inside an existing internal vesicle (example 2, one observation). **d** Protoplasts and S-cells of *K. viridifaciens* pIJ82-GFP incubated with D-TR for 72 h. Note that no internalization of D-TR was observed (at least 100 protoplasts and 10 S-cells from one experiment).

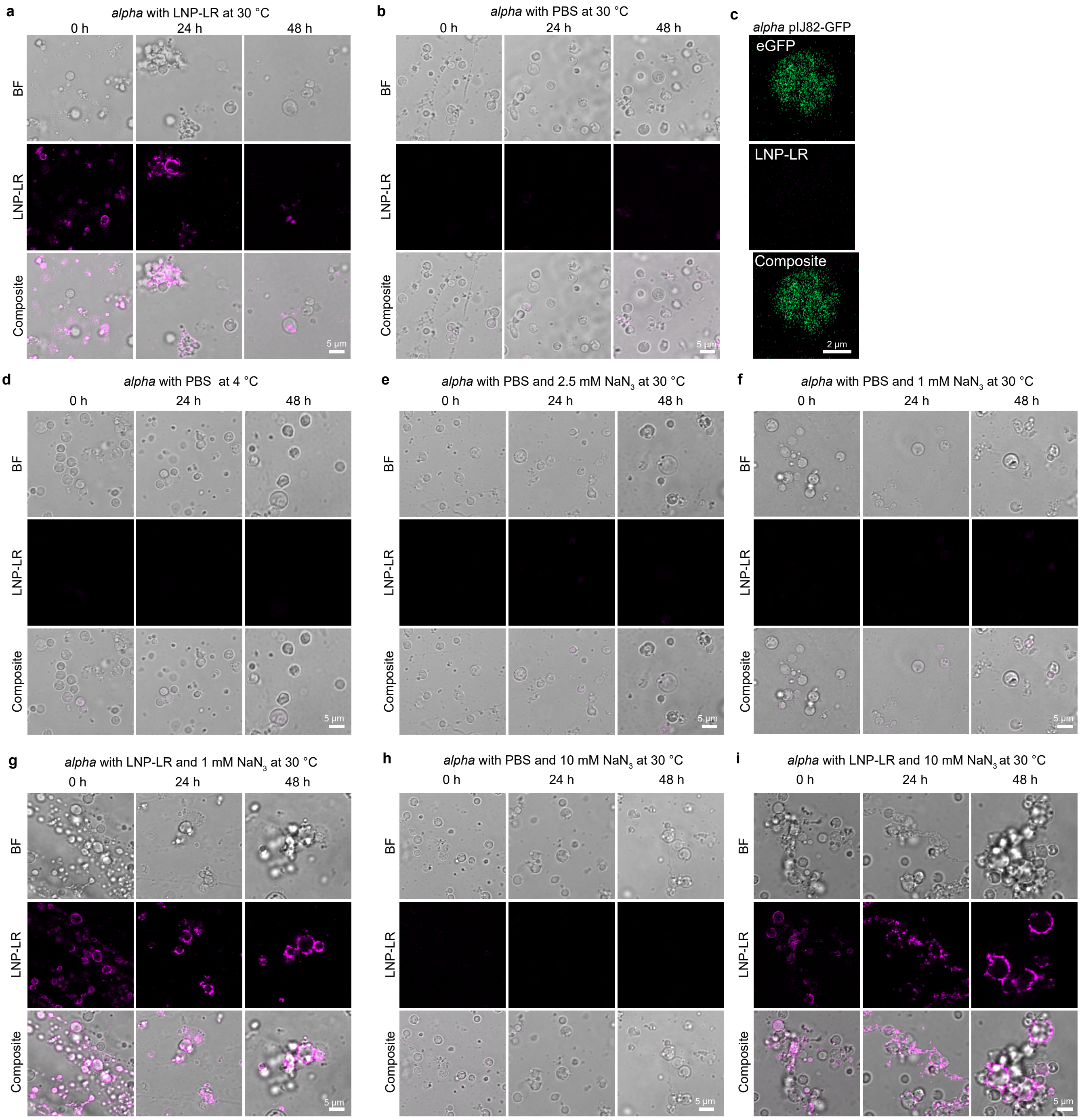

Supplementary Fig. 4: Uptake of LNP-LR by *alpha*.

a, b *alpha* incubated with LNP-LR (LNP-Liss Rhod; magenta) (**a**) or PBS (Phosphate Buffered Saline) (**b**) at 30 °C showing localization of LNP-LR after 0, 24 and 48 h and examples of autofluorescence, respectively. **c** *alpha* pIJ82-GFP incubated for 3 days without LNP-LR as imaging control for Fig. 3b (one experiment with three control cells). **d**-**e** *alpha* incubated with PBS at 4 °C (**d**) or at 30 °C in the presence of 2.5 mM sodium azide (NaN_3) (**e**) as control for fluorescence emission of cells in Fig. 3d and e, respectively. (**f**-**i**) *alpha* incubated with PBS or LNP-LR at 30 °C with 1 or 10 mM sodium azide as indicated. Images (**a**-**b**, **d**-**i**), were obtained after 0-, 24-and 48-h incubation from one experiment.

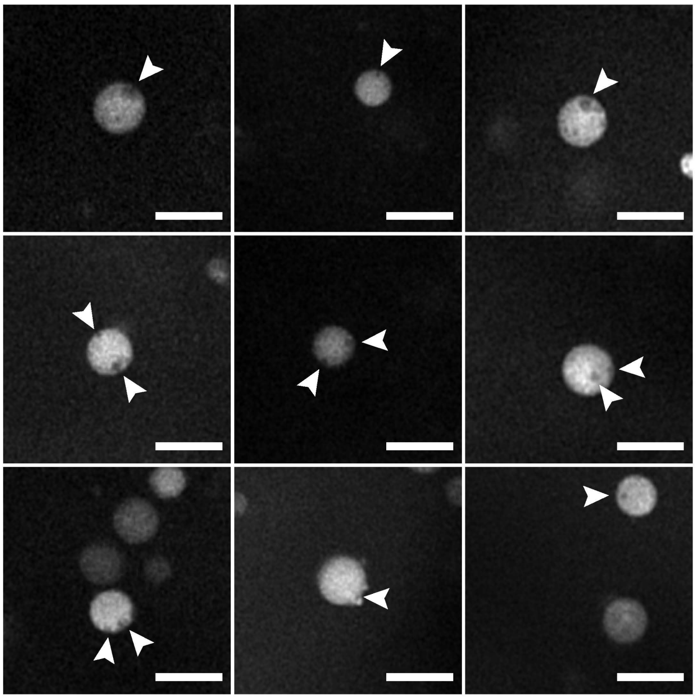

Supplementary Fig. 5. High resolution cryo-fluorescence of L-forms.

Examples of *alpha* pIJ82-GFP cells (*n* = 9) imaged using cryo-fluorescence microscopy (eGFP emission depicted in grey), originating from four regions of interest (from a total of 6). Putative vesicles are indicated with arrows. Images were captured using the long distance 100x objective. Scale bars indicate 5 µm.

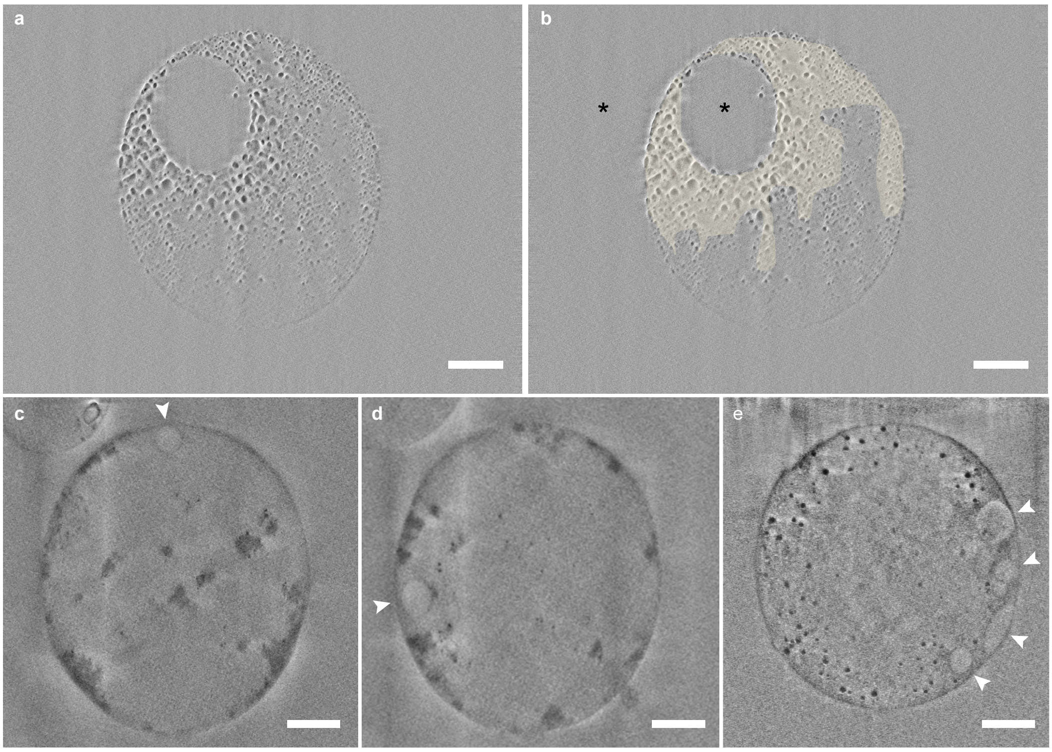

Supplementary Fig. 6. Over-dose experiment of L-form cell using FIB-SEM and L-form vesicles.

a-b FIB-SEM slice of over-dose experiment using *alpha* pIJ82-GFP (performed for one cell). The yellow colour in (**b**) indicates areas with distinguished beam damage. The vesicle (indicated by black asterisk inside the cell) seems to be less to none affected by the over-dose, similar to the medium outside the cell (black asterisk outside of the cell). The image in Fig. 4d was taken before this experiment, and Fig. 4e is obtained by summing several slices deeper in the cell after acquiring this image. Scale bar indicates 1 µm.
c-e FIB-SEM slices of two cells in which (**c-d**) correspond to the cell in Fig. 4f and (**e**) corresponds to the cell in Fig. 4 h-k). White arrows indicate vesicles that line the cell membrane (three slices of two imaged cells shown). Scale bar in (**c-e**) indicates 500 nm.

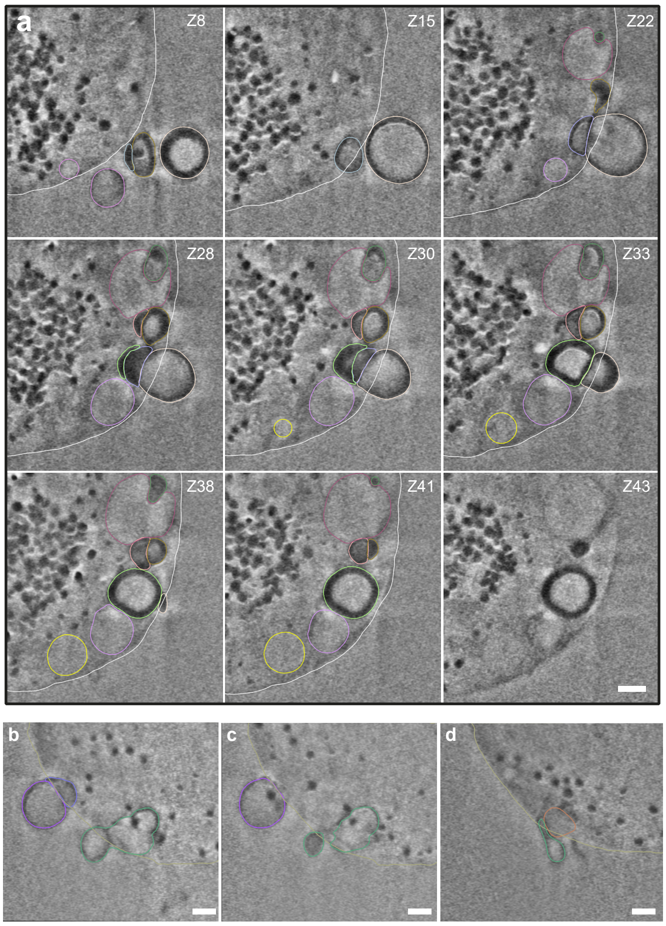

Supplementary Fig. 7. 3D Segmentation of L-form vesicles.

a FIB-SEM slices corresponding to the cell in Fig. 4h. Z-number indicates the slice. Colours indicate individual vesicles. See also Supplementary Movie 3.
b-d FIB-SEM slices corresponding to Fig. 4i-q, respectively. Colours correspond to the segmented colours in Fig. 4m, q. Vesicles that are budding out the cells are connected to other vesicles or are elongated inside the cell. See also Supplementary Movie 4. 3D segmentation as shown in (a-d) was performed for two different regions of one cell. Scale bars indicate 200 nm.

Supplementary Tables

Supplementary Table 1. Overview of protein BLAST results. Significant hits (gene locus tag given) (E-value < 1e-06), E-value and percent identity of the indicated protein sequences used as query against *K. viridifaciens* DSM40239. See Supplementary Data 1 for the full query protein sequences and accession numbers.

Organism	Protein	Hits	E-value	Percent identity
Bacillus subtilis strain 168	ComEA	BOQ63_029625	5.28e-24	34.90%
	ComEC	BOQ63_029630	1.78e-09	26.81%
	ComFA	BOQ63_020315	1.03e-07	24.83%
	ComGA, ComGB, ComGC, ComGD, ComGE, ComGF, ComGG, ComC	-	-	
Neisseria gonorrhoeae	ComE	BOQ63_029625	4.05e-10	46.43%
	ComA, ComP*, PilC, PilD, PilE1, PilV, PilQ, PilF, PilG, PilT	-	-	
Helicobacter pylori strain P12	ComE3,	-	-	
	ComB2-4, Com6-10			

*Protein sequence obtained from Wolfgang, van Putten[1]

Supplementary Table 2. Quantification of internal vesicles. Quantification of the percentage of 7-day-old L-forms (*alpha* pRed*), freshly harvested S-cells and protoplasts (*K. viridifaciens* pRed*) with internal vesicles after 0- or 3-day incubation with SynapseRed and stained with SYTO 9. Integer numbers indicate the number of cells with or without internal vesicles. The percentage of cells with putative internal vesicles is given (positive %).

	0-day incubation				3-day incubation			
Cell type	Vesicle	No vesicle	Total	Positive (%)	Vesicle	No vesicle	Total	Positive (%)
L-forms	50	154	204	24.51	37	215	252	14.68
S-cells	5	218	223	2.24	9	337	346	2.60
Protoplasts	1	203	204	0.49	1	237	238	0.42

Supplementary Table 3. Quantification of Dextran-Texas Red uptake. Quantification of the percentage of cells that take up Dextran-Texas Red (D-TR) after a 3-day incubation (positive %). 7-day-old L-forms (*alpha* pIJ82-GFP), S-cells and protoplasts (*K. viridifaciens* pIJ82-GFP) were incubated in duplo (replica 1 and 2) with Phosphate Buffered Saline (PBS, control) or D-Texas Red. The number of cells with or without uptake of D-TR are indicated.

Cell type	Condition	Replica	Uptake	No uptake	Total cells counted	Positive (%)
L-forms	PBS	1	0	101	101	0.0
		2	0	602	602	0.0
	D-TR	1	18	284	302	6.0
		2	11	176	187	5.9
S-cells	PBS	1	0	75	75	0.0
		2	2	94	96	2.1
	D-TR	1	5	295	300	1.7
		2	1	114	115	0.9
Protoplasts	PBS	1	0	168	168	0.0
		2	1	127	128	0.8
	D-TR	1	2	186	188	1.1
		2	0	79	79	0.0

Supplementary Table 4. Strains used in this study.

Strain	Description	Notes/references
Escherichia coli JM109	*recA*1, *endA*1, *gyrA*96, *thi*, *hsdR*17, *supE*44, *relA*1, λ⁻, Δ (*lac-proAB*), [F', *traD*36 *proAB, lacI*^{q}ZΔM15]	[2]
Escherichia coli ET12567/pUZ8002	Methylation-deficient strain<br>(F⁻, *dam*-13::*Tn*9, *dcm*-6, *hsdM*, *hsdR*, *recF*143, *zjj*-202::Tn10, *galK*2, *galT*22, *ara*14, *lacY*1, *xyl*-5, *leuB*6, *thi*-1, *tonA*31, *rpsL*136, *hisG*4, *tsx*-78, *mtl*-1, *glnV*44) carrying the non-transmissible pUZ8002 plasmid	[3]
Kitasatospora viridifaciens DSM40239	Wild-type strain	[4]
alpha	L-form derivative of DSM40239 obtained after exposure to Penicillin G and lysozyme	[5]
M1	L-form derivative of DSM40239 obtained after exposure to hyperosmotic stress	[5]
delta	L-form derivative of DSM40239 obtained after exposure to Penicillin G and lysozyme	[6]
alpha pIJ82-GFP	*alpha* containing pIJ82-GFP	This work
alpha pKR2	*alpha* containing pKR2, which contains a C-terminal eGFP gene fusion to *divIVA* under the control of the *Streptomyces coelicolor gap1* promoter	[7]
*alpha*Δ*divIVA*	*divIVA::aac(3)IV*	[7]
*alpha*Δ*divIVA* pIJ82-GFP	*alpha*Δ*divIVA* containing pIJ82-GFP	This work
*alpha*Δ*ssgB*	*ssgB::aac(3)IV*	[5]
*alpha*Δ*comEA/EC*	*(comEA-comEC)::aac(3)IV*	This work

Supplementary Table 5. Plasmids used in this study.

Plasmid	Features	Notes/References
pRed*	pIJ8630-derivative expressing *mCherry* under control of the *S. coelicolor A3(2) gap1* promoter	[8]
pGreen	pIJ8630-derivative expressing *eGFP* under control of the *S. coelicolor A3(2) gap1* promoter	[9]
pSET152	*E. coli*-*Streptomyces* shuttle vector; high copy number in *E. coli* and integrating in the ϕC31 *attB* site in *Streptomyces*	[10]
pIJ82	pSET152-derivative carrying a hygromycin resistance cassette	Kindly provided by Dr. B. Gust (JIC)
pIJ82-GFP	pSET152-derivative expressing *eGFP* under control of the *S. coelicolor gap1* promoter	This work
pMS82	*E. coli*-*Streptomyces* shuttle vector integrative in the ϕBT1 *attB* site for genomic integration in *Streptomyces*	[11]
pWHM3-oriT	Self-replicating, multi-copy, unstable plasmid harboring *oriT,* used as *E. coli/Streptomyces* shuttle vector	[12]
pWHM3-oriT-hyg	pWHM3-oriT-derivative carrying a hygromycin resistance cassette inserted in to the *tsr* gene in the EcoRV site	This work
pFL-*ssgB*	pWHM3-oriT-hyg-derivative containing a hygromycin resistance gene and a downstream flanking sequence of *ssgB* downstream derived from pKR1 to enable integration into the genome	This work
pRK1	pWHM3-oriT containing both flanks of the *comEA*-*comEC* region interspersed with the *apra*-*loxP* cassette conferring resistance to apramycin	This work
pKR1	pWHM3-based construct used to replace *ssgB* by *aac(3)IV*	[5]
pKR2	pIJ8630 derivative carrying a viomycin resistance cassette and expressing a C-terminal *eGFP* fusion to *divIVA* under control of the *S. coelicolor* A3(2) *gap1* promoter	[7]

Supplementary Table 6. Primers used in this study.

Primer	Sequence (5' - 3')	Notes	Reference
Hyg_F-231_EEV	ctgaGAATTCGATATCGATCGGCGGGGCCTGGCGGCG	Amplification of the hygromycin resistance cassette from pMS82	This work
Hyg_R+1237_HEV	ctgaAAGCTTGATATCGGATCCTTGCCGAGCTGGGAT	Amplification of the hygromycin resistance cassette from pMS82	This work
FL1-comEA/comEC-FW	GACGAATTCAGGACCGGATGCACCGGTTC	Amplification of flank 1 of *comEA-comEC* locus	This work
FL1-comEA/comEC-REV	GAATCTAGACCGCACCGTCTCGTTGATCG	Amplification of flank 1 of *comEA-comEC* locus	This work
ComEA_Apra_check_FW	CACTCGTGTGAGTGACCGTT	Amplification of *comEA* region in PCR1 mix	This work
ComEC_Apra_check_RV	AACGGCAAGGGTGGACG	Amplification of *comEA* region in PCR1 mix	This work
ComEC_Presence_Check_1_FW	TACGACACCGAGTCCGCAG	Amplification of *comEC* region 1 in PCR2 mix	This work
ComEC_Presence_Check_1_RV	CGCAAGGGCCAACATGTCTC	Amplification of *comEC* region 1 in PCR2 mix	This work
ComEC_Presence_Check_2_FW	AGACCCTCCTCACCGTCAAG	Amplification of *comEC* region 2 in PCR3 mix	This work
ComEC_Presence_Check_2_RV	GACAGCAGGAAACCGAAGGA	Amplification of *comEC* region 2 in PCR3 mix	This work
gap1_FW_BglII	GATTACAGATCTCCGAGGGCTTCGAGACC	Amplification of the region containing the *gap1* promoter and *eGFP* gene from pGreen	This work
egfp_RV_EcoRI	TAAGCAGAATTCTTACTTGTACAGCTCGTCCA	Amplification of the region containing the *gap1* promoter and *eGFP* gene from pGreen	This work
SsgB_Presence_FW	GGCGGGTACTCCGTGATGATTC	Confirmation of *ssgB* replacement by *apra-loxP* cassette	This work
SsgB_Presence_RV	AGCTTTCGGCGAGGATGTGG	Confirmation of *ssgB* replacement by *apra-loxP* cassette	This work
Tsr_Hyg_FW1	AAGGCCAAGACATTCGGCAT	Confirmation of presence of pFL-*ssgB* in natural transformants	This work
Tsr_Hyg_RV1	CGAGCGACGTGCGTACTATC	Confirmation of presence of pFL-*ssgB* in natural transformants	This work

Supplementary Table 7. Imaging settings used with the Zeiss LSM 900 confocal microscope.

Fluorescent protein, dye or particle	Excitation (nm)	Emission filter (nm)
eGFP	488	490-575
mCherry	561	565-700
SYTO 9	488	490-575
SynapseRed C2M	488	571-700
Dextran-Texas Red	561	560-700
Cy5	640	450-700
LNP-LR	561	565-700

Supplementary Table 8. Characterization of lipid nanoparticles. Dynamic Light Scattering (DLS) and ζ-potential of lipid nanoparticles. PDI = polydispersity index. Source data are provided as a Source Data file.

LNP	Avg. size (nm) at 25 °C	PDI	ζ-potential (mV) at 25 °C
LNP-LR	151.1	0.144	-8.52

Supplementary References

1. Wolfgang, M., van Putten, J. P. M., Hayes, S. F. & Koomey, M. The *comP* locus of *Neisseria gonorrhoeae* encodes a type IV prepilin that is dispensable for pilus biogenesis but essential for natural transformation. *Mol. Microbiol.* **31**, 1345-1357 (1999).

2. Yanisch-Perron, C., Vieira, J. & Messing, J. Improved M13 phage cloning vectors and host strains: nucleotide sequences of the M13mp18 and pUC19 vectors. *Gene* **33**, 103-119 (1985).

3. MacNeil, D. J., Gewain, K. M., Ruby, C. L., Dezeny, G., Gibbons, P. H. & MacNeil, T. Analysis of *Streptomyces avermitilis* genes required for avermectin biosynthesis utilizing a novel integration vector. *Gene* **111**, 61-68 (1992).

4. Ramijan, K., van Wezel, G. P. & Claessen, D. Genome sequence of the filamentous actinomycete *Kitasatospora viridifaciens*. *Genome Announc.* **5**, e01560-01516 (2017).

5. Ramijan, K. *et al.* Stress-induced formation of cell wall-deficient cells in filamentous actinomycetes. *Nat. Commun.* **9**, 5164 (2018).

6. Shitut, S. *et al.* Generating heterokaryotic cells via bacterial cell-cell fusion. *Microbiol. Spectr.*, e01693-22 (2022).

7. Zhang, L. *et al.* An alternative and conserved cell wall enzyme that can substitute for the lipid II synthase MurG. *mBio* **12**, 03381-20 (2021).

8. Zacchetti, B., Smits, P. & Claessen, D. Dynamics of pellet fragmentation and aggregation in liquid-grown cultures of *Streptomyces lividans*. *Front Microbiol* **9**, 943 (2018).

9. Zacchetti, B. *et al.* Aggregation of germlings is a major contributing factor towards mycelial heterogeneity of *Streptomyces*. *Sci. Rep.* **6**, 27045 (2016).

10. Bierman, M., Logan, R., O'Brien, K., Seno, E. T., Rao, R. N. & Schoner, B. E. Plasmid cloning vectors for the conjugal transfer of DNA from *Escherichia coli* to *Streptomyces* spp. *Gene* **116**, 43-49 (1992).

11. Gregory, M. A., Till, R. & Smith, M. C. M. Integration site for *Streptomyces* phage phiBT1 and development of site-specific integrating vectors. *J. Bacteriol.* **185**, 5320-5323 (2003).

12. Wu, C. *et al.* Lugdunomycin, an angucycline-derived molecule with unprecedented chemical architecture. *Angew. Chem. Int. Ed. Engl.* **58**, 2809-2814 (2019).
