# Supplementary material for: Endocytosis-like DNA uptake by cell wall-deficient bacteria
Source: Nat Commun. 2022 Sep 22;13:5524. doi: 10.1038/s41467-022-33054-w (PMC9500057; doi:10.1038/s41467-022-33054-w)
Supplement: Supplementary file 2 — Description of Additional Supplementary Files [file 41467_2022_33054_MOESM2_ESM.pdf]

Title: Supplementary Movie 1:
Description: 3D reconstruction of DNA localization, related to Fig. 2a.
3D reconstruction of the cell in Fig. 2a showing localization of Cy-5 labelled plasmid DNA (pFL-*ssgB*; magenta) in an internal vesicle of *alpha* pIJ82-GFP (cytoplasmic eGFP; green) as generated using the 3D Viewer plugin in Fiji using standard settings (ImageJ).

Title: Supplementary Movie 2:
Description: Uptake of Dextran-Texas Red by L-forms, related to Fig. 2e
Timelapse video of *alpha*-DivIVA-eGFP (green) incubated with 3 kDa Dextran-Texas Red (D-TR; magenta). Left: Brightfield. Right: Composite of green and magenta channels. Scale bar indicates 1 μm.

Title: Supplementary Movie 3:
Description: 3D Reconstruction of vesicles in L-form cell, related to Fig. 4
3D segmentation volume rendering of vesicles of *alpha* pIJ82-GFP based on FIB-SEM Z-stack slices corresponding to Fig. 4h and Supplementary Fig. 7a. Colours indicate individual vesicles or vesicle complexes. The cell is depicted in grey in the 3D Volume Rendering.

Title: Supplementary Movie 4:
Description: 3D Reconstruction of vesicles in L-form cell, related to Fig. 4
3D segmentation volume rendering of vesicles of *alpha* pIJ82-GFP based on FIB-SEM Z-stack slices corresponding to Fig. 4i-q and Supplementary Fig. 7b-d. Colours indicate individual vesicles or vesicle complexes. The cell is depicted in grey in the 3D Volume Rendering.

Title: Supplementary Movie 5:
Description: Timelapse of putative vesicle disruption.
Timelapse of *alpha* pRed* expressing cytoplasmic mCherry, showing putative vesicle disruption between 01:00 to 01:15 h:min of incubation. The vesicle was present from the start of the incubation. Cells were incubated at 30 °C and imaged overnight using the Lionheart FX automated microscope. Images were taken every 15 min for 20 h. Left: Brightfield. Right: mCherry emission.

Title: Supplementary Movie 6:
Description: Timelapse of putative vesicle disruption.
Timelapse of *alpha* pRed* expressing cytoplasmic mCherry, showing putative vesicle disruption between 13:30 to 13:45 h:min of incubation. The vesicle was present from the start of the incubation. Cells were incubated at 30 °C and imaged overnight using the Lionheart FX automated microscope. Images were taken every 15 min for 17.5 h. Left: Brightfield. Right: mCherry emission.

Title: Supplementary Movie 7:
Description: Timelapse of putative vesicle disruption.
Timelapse of *alpha* pRed* expressing cytoplasmic mCherry, showing putative vesicle disruption between 17:15 to 17:30 h:min of incubation, after which the timelapse had ended. Note that the movie shows the incubation from 15 h onwards. The vesicle was present from the start of the incubation. Cells were incubated at 30°C and imaged overnight using the Lionheart FX automated microscope. Images were taken every 15 min for 17.5 h. . Left: Brightfield. Right: mCherry emission.

Title: Supplementary Data 1:
Description: Protein sequences used for protein BLAST.
List of protein sequences in FASTA format with accession numbers, used to perform the protein BLAST search against *K. viridifaciens* DSM40239. Note that the protein sequence of ComP was obtained from literature as stated in the sequence header.
