# Supplementary material for: Endocytosis-like DNA uptake by cell wall-deficient bacteria
Source: Nat Commun. 2022 Sep 22;13:5524. doi: 10.1038/s41467-022-33054-w (PMC9500057; doi:10.1038/s41467-022-33054-w)
Supplement: Supplementary file 11 — Reporting Summary [file 41467_2022_33054_MOESM11_ESM.pdf]

nature portfolio

Corresponding author(s): Dennis Claessen & Gilles P. van Wezel

Last updated by author(s): Aug 27, 2022

Reporting Summary

Nature Portfolio wishes to improve the reproducibility of the work that we publish. This form provides structure for consistency and transparency in reporting. For further information on Nature Portfolio policies, see our Editorial Policies and the Editorial Policy Checklist.

Statistics

For all statistical analyses, confirm that the following items are present in the figure legend, table legend, main text, or Methods section.

n/a	Confirmed	
☐	☒	The exact sample size (*n*) for each experimental group/condition, given as a discrete number and unit of measurement
☐	☒	A statement on whether measurements were taken from distinct samples or whether the same sample was measured repeatedly
☐	☒	The statistical test(s) used AND whether they are one- or two-sided<br>*Only common tests should be described solely by name; describe more complex techniques in the Methods section.*
☒	☐	A description of all covariates tested
☐	☒	A description of any assumptions or corrections, such as tests of normality and adjustment for multiple comparisons
☐	☒	A full description of the statistical parameters including central tendency (e.g. means) or other basic estimates (e.g. regression coefficient) AND variation (e.g. standard deviation) or associated estimates of uncertainty (e.g. confidence intervals)
☐	☒	For null hypothesis testing, the test statistic (e.g. *F*, *t*, *r*) with confidence intervals, effect sizes, degrees of freedom and *P* value noted<br>*Give P values as exact values whenever suitable.*
☒	☐	For Bayesian analysis, information on the choice of priors and Markov chain Monte Carlo settings
☒	☐	For hierarchical and complex designs, identification of the appropriate level for tests and full reporting of outcomes
☒	☐	Estimates of effect sizes (e.g. Cohen's *d*, Pearson's *r*), indicating how they were calculated

Our web collection on statistics for biologists contains articles on many of the points above.

Software and code

Policy information about availability of computer code

Data collection	Protein homology search was performed using the NCBI offline BLAST software (v. 2.12.0).<br>Fluorescence microscopic images were acquired with Zeiss Zen 3.1 software (Blue Edition)<br>Software for the Lionheart FX automated microscope (BioTek) with Gen 5 v. 3.10.<br>Stereomicroscopy images were taken using Bresser MikroCamLab II software Version: x64, 4.7.15283.20190804<br>Colony plates were scanned using the Epson Perfection V600 Photo scanner with Epson Scan Utility v3.9.2.0 software.<br>Fluorescent images from high-pressure-frozen samples were imaged using Zeiss software version 3.1<br>Fluorescence Microscopy images were processed using the 'Plot profile', Cell Counter' or 'Calculate GP' plugin in Fiji (ImageJ) where applicable, latest Fiji version being v1.53s.<br>Laurdan dye fluorescence emission in the 96-wells plate assay was measured using the Spark® multimode microplate reader (Tecan) with Sparkcontrol V3.1 software.

Data analysis	Statistics were performed using SPSS statistics software (IBM, version 27.0).<br>Cryo-FIB-SEM images were processed using MATLAB (R2018b, Natick, Massachusetts: The MathWorks Inc.)<br>Fluorescence Microscopy images were processed using Fiji (ImageJ), latest version being v1.53s.<br>Graphs were plotted using Graphpad Prism v 9.0.0 or using R version 3.6.1<br>3D segmentation was performed using the DragonflyTM imag analysis software version 2021.1.<br>Graphical images were generated using Adobe Illustrator v. 26.3.1 or via BioRender.com, the latter accessed in August 2022.

For manuscripts utilizing custom algorithms or software that are central to the research but not yet described in published literature, software must be made available to editors and reviewers. We strongly encourage code deposition in a community repository (e.g. GitHub). See the Nature Portfolio guidelines for submitting code & software for further information.

Data

Policy information about availability of data

All manuscripts must include a data availability statement. This statement should provide the following information, where applicable:

- Accession codes, unique identifiers, or web links for publicly available datasets
- A description of any restrictions on data availability
- For clinical datasets or third party data, please ensure that the statement adheres to our policy

Data availability statement as in Manuscript:
Data underlying all graphs, Fig. 1b, Supplementary Fig. 1a, Supplementary Fig. 1c and Supplementary Table 8 is provided in the Source Data file. Protein sequences obtained from the UniProt database or literature to perform the NCBI BLAST search are provided with accession numbers in Supplementary Data 1. All fluorescence and FIB-SEM micrographs in this paper, including the raw FIB-SEM data underlying the 3D segmentation volume rendering (Supplementary Movie 3 and 4), as well as the micrographs used for vesicle and D-TR uptake quantification in Fig. 3f and Supplementary Table 2 and 3, have been deposited in the Open Science Framework (OSF) database available at https://doi.org/10.17605/OSF.IO/5WKGJ. For the BlastP search, hits were collected from Streptomyces viridifaciens strain DSM40239 with accession numbers CP090840 (https://www.ncbi.nlm.nih.gov/nuccore/CP090840), CP090841(https://www.ncbi.nlm.nih.gov/nuccore/CP090841) and CP090842 (https://www.ncbi.nlm.nih.gov/nuccore/CP090842). Streptomyces viridifaciens ATTC11989 (accession CP023698, https://www.ncbi.nlm.nih.gov/nuccore/CP023698/) was used to deduce the putative K. viridifaciens comEC start and stopcodon for the gene knockout construct. Source data are provided with this paper.

Human research participants

Policy information about studies involving human research participants and Sex and Gender in Research.

Reporting on sex and gender	N/A
Population characteristics	N/A
Recruitment	N/A
Ethics oversight	N/A

Note that full information on the approval of the study protocol must also be provided in the manuscript.

Field-specific reporting

Please select the one below that is the best fit for your research. If you are not sure, read the appropriate sections before making your selection.

☒ Life sciences ☐ Behavioural & social sciences ☐ Ecological, evolutionary & environmental sciences

For a reference copy of the document with all sections, see nature.com/documents/nr-reporting-summary-flat.pdf

Life sciences study design

All studies must disclose on these points even when the disclosure is negative.

Sample size	Sample sizes are stated in the main text figure legend or methods section and were based on standards in the field of microbiology. For example, transformation experiments to compare strains or conditions consisted of 3 - 5 replicates as used in published studies (https://doi.org//10.1128/JB.00633-13, https://doi.org/10.1007/s00253-012-3987-2). Membrane fluidity (plate assay) was based on three biological and three technical measurements as stated in https://www.ncbi.nlm.nih.gov/pmc/articles/PMC8342135/ (doi: 10.21769/BioProtoc.3063). Sample sizes were based on standards in the field and thus no sample size calculation was performed.
Data exclusions	No data was excluded
Replication	Replicates for all experiments are mentioned in the figure legends.<br>For all DNA uptake and membrane fluidity experiments, biological replicates always originated from separate bacterial cultures to ensure reproducibility.

	Imaging with dyes and uptake of LNP-LR or D-TR was performed for multiple separate experiments which showed consistent results (e.g. Fig. 3a, 3b and Supl. 4a show uptake of LNP-LR in separate experiments).<br>Micrographs show representative images of multiple similar observations per experiment, the nr of observations are listed in the legends. Cryo FIB-SEM was performed on several multiple frozen cell samples, all replicates resulted in similar results.<br>Two membrane fluidity experiments, with slightly different results, are both given in Fig. 1e and Supplementary Fig. 2a.<br>Imaging of the engulfment of Cy-5 DNA (Fig. 2a), as well as the timelapse of D-TR uptake in alpha pKR2 (Fig. 2e) was not repeated as the occurence was low and served only to show that extracellular DNA or D-TR has the ability to end up inside internal L-form vesicles, without making further statements on the frequency of this event. In addition, the frequency of D-TR uptake was quantified in Supplementary Table 3 for >170 cells per cell type.<br>Spontaneous transformation for L-form M1 (Fig. 1b) yielded numerous transformants at the first attempt and was therefore not repeated (only served to show this cell type can take up external plasmid DNA). PEG-transformation with gDNA (Supl. Fig 1a) was repeated for two cell culture ages that gave the same result . PEG-based transformation for different DNA types (Supl. Fig. 1c) was performed once for each DNA type as the ability of the cells to become transformed with this DNA was successful at the first attempt and gave many transformants (For methylated DNA, it is common knowledge that unmethylated plasmid DNA should be used to transform Streptomycete-like bacteria, see Kieser, T., Bibb, M. J., Buttner, M. J., Chater, K. F., & Hopwood, D. A. (2000). Practical streptomyces genetics (Vol. 291, p. 397). Norwich: John Innes Foundation.)
Randomization	Agar plates with solid medium were randomized before being assigned to usage for different strains. For cell counting quantifications, all cells in view were imaged (except the ones that were not laying still) instead of specific cells. Locations for representative microscopy imaging for quantification was performed on multiple random locations of the sample using the 'tiles' option or by imaging multiple successive locations. No other randomization was performed as, for the given experiments, this is not required for the experimental design.
Blinding	Blinding was not used. For quantification, the 'Plot Profile' tool in ImageJ was used to confirm or reject the presence of internal vesicles or uptake of D-TR.

Reporting for specific materials, systems and methods

We require information from authors about some types of materials, experimental systems and methods used in many studies. Here, indicate whether each material, system or method listed is relevant to your study. If you are not sure if a list item applies to your research, read the appropriate section before selecting a response.

Materials & experimental systems

n/a	Involved in the study
☒	☐ Antibodies
☒	☐ Eukaryotic cell lines
☒	☐ Palaeontology and archaeology
☒	☐ Animals and other organisms
☒	☐ Clinical data
☒	☐ Dual use research of concern

Methods

n/a	Involved in the study
☒	☐ ChIP-seq
☒	☐ Flow cytometry
☒	☐ MRI-based neuroimaging
